# Carboxyl‐Guided Ultrafine Zinc‐Acetate Templating Enables Closed‐Pore Engineering in Coal‐Derived Hard Carbon Anodes for High‐Performance Sodium‐Ion Batteries

**DOI:** 10.1002/advs.202512483

**Published:** 2025-10-13

**Authors:** Jialiang Yuan, Guokan Liu, Chi Wang, Fang Wan, Lang Qiu, Bo Yuan, Tingru Chen, Zhenguo Wu

**Affiliations:** ^1^ School of Chemical Engineering Sichuan University Chengdu 610065 P. R. China; ^2^ Ordos Carbon Neutral Research and Application Co., Ltd Ordos 017010 P.R. China

**Keywords:** bituminous coal, chelation, closed pores, hard carbon, structural control

## Abstract

Coal‑derived hard carbon (HC) is an appealing anode for sodium‑ion batteries (SIBs) thanks to its abundance and cost‑effectiveness, yet its practical deployment is hampered by modest capacity and a low initial coulombic efficiency (ICE) stemming from uncontrolled porosity. Here, carboxyl‑mediated chelation chemistry is combined with an ultrafine zinc acetate (ZA) templating (≈2 nm) approach and phenolic‑resin interfacial encapsulation to deliver deterministic closed‑pore engineering in bituminous‑coal HC. NaOH activation generates a carboxyl‑rich surface that homogeneously anchors ZA nanodomains. Resin coating locks these templates at the coal/resin interface, and a sequence of mild oxidation, gradient carbonization, and acid leaching yields a dense network of closed nanocavities. The optimized HC delivers 370.9 mAh g^−1^ at 20 mA g^−1^, retains 92.5% ICE, and sustains 80.4% of its capacity over 3000 cycles at 1 A g^−1^, surpassing state‑of‑the‑art coal‑based HCs. These findings illustrate a scalable, molecular‑level strategy that reconciles low‑cost feedstocks with high‑performance SIB anodes.

## Graphical abstract

In this study, ≈2 nm zinc acetate templates are uniformly anchored on the surface of NaOH‐etched bituminous coal via chelation between carboxyl groups and Zn^2+^. Subsequently, phenolic resin encapsulation is employed to construct a hard carbon material with abundant closed‐pore structures, achieving a reversible capacity of 370.9 mAh g^−1^ and an ICE of 92.5%.

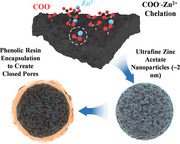

## 1 Introduction

The geological abundance of sodium (2.36 wt.% in Earth's crust) and its cost‐effective extraction profile position sodium‐ion batteries (SIBs) as pivotal alternatives to lithium‐ion systems in sustainable energy storage applications.^[^ ^1^, ^2^, ^3^, ^4^ ^]^ Nevertheless, the development of commercially viable SIBs is fundamentally limited by the inadequate performance metrics of current carbon‐based anodes.^[^ ^5^, ^6^, ^7^, ^8^ ^]^

Hard carbon (HC) has emerged as the prevailing anode material for SIBs, with its disordered turbostratic carbon layers and expanded interlayer spacing synergistically facilitating efficient sodium‐ion intercalation.^[^ ^9^, ^10^ ^]^ At present, precursor selection and microstructural control are the core research directions. Biomass (e.g., coconut shells and straw), synthetic polymers (e.g., phenolic resins), and fossil fuel derivatives (e.g., asphalt and coal) constitute the three mainstream precursor categories that have dominated recent research efforts.^[^ ^11^, ^12^, ^13^, ^14^ ^]^ However, HC anodes exhibit generally low initial coulombic efficiency (ICE) (60%–80%) accompanied by significant capacity decay at high rates, while the structure‐property relationship between HC's microstructure and its sodium storage performance remains insufficiently elucidated. These limitations severely impede HC material development. Consequently, enhancing HC's comprehensive performance through precursor modification and microstructure optimization represents the primary focus of current research.^[^ ^15^, ^16^, ^17^ ^]^

Coal (e.g., anthracite and bituminous coal) has abundant reserves, high carbon yield, and low cost, and is not limited by seasons or regions. Its supply chain is stable, making it well‐suited for large‐scale, continuous production.^[^ ^18^, ^19^ ^]^ As a cornerstone of traditional energy systems, the high‐value utilization of coal through advanced material engineering has emerged as a strategic research priority in the global energy transition landscape. Compared to biomass/resin‐based counterparts, coal‐derived HC demonstrates superior economic viability, yet suffers from critical limitations, including specific capacities below 300 mAh g^−1^, poor ICE, and inferior cycling stability.^[^ ^20^ ^]^ Therefore, it is necessary to improve its electrochemical performance through modification methods. Current research indicates that the microstructure and electrochemical properties of coal‐based HC can be effectively optimized by adjusting carbonization temperature (1000–1500 °C), introducing activators (KOH, CO_2_), or heteroatom doping (N, S, P).^[^ ^21^, ^22^, ^23^ ^]^ For example, Su et al.^[^ ^24^ ^]^ enhanced the d002 interlayer spacing of carbonized HC through simple pre‐oxidation, increased defect sites, and prepared HC with a reversible capacity of 306.3 at 30 mAh g^−1^. Fu et al.^[^ ^25^ ^]^ mixed coal with KOH and annealed it at 400 °C to remove minerals and activate precursors, establishing abundant closed nanopores on the surface of HC. The optimized HC delivered a specific capacity of 328.5 mAh g^−1^ and an ICE of 92.2%. In addition, the template method is an effective strategy for regulating the closed‐pore structure of HC. For instance, Wen et al.^[^ ^26^ ^]^ constructed size‐controllable ZnO microcrystals in the precursor through a zinc salt template and induced the formation of a large number of closed pores during the subsequent pyrolysis process. However, the inherent structural complexity of coal precursors—including cross‐linked aromatic clusters, irregularly distributed mineral inclusions, as well as issues such as uneven template dispersion in complex structures and weak interface interaction with the carbon matrix—severely restricts the precise control of their microstructure, posing a fundamental technical challenge.^[^ ^27^ ^]^ This persistent limitation highlights the critical demand for innovative synthesis approaches that simultaneously boost electrochemical performance and enable scalable production of coal‐based HC anodes.

Inspired by coordination chemistry and interfacial polymer science, this work proposes a “chelation–encapsulation–carbonization” three‐stage strategy. Initially, carboxyl functional groups are immobilized on the surface of bituminous coal via NaOH etching. Through the chelation between carboxyl groups and Zn^2+^ ions, an ultrafine zinc acetate (ZA) nano‐template (≈2 nm) is in situ formed. Subsequently, phenolic resin interfacial encapsulation, pre‐carbonization, acid leaching, and high‐temperature carbonization are conducted, ultimately yielding a HC material with a high‐density closed‐pore architecture. By precisely tuning the ZA loading, a balance is achieved between a low specific surface area (11.9 m^2^ g^−1^) and an ultrahigh closed‐pore surface area (551.5 cm^2^ g^−1^). The resulting HC anode demonstrates a reversible specific capacity of 370.9 mAh g^−1^, an ICE of 92.5%, and excellent cycling stability, maintaining 80.4% of its initial capacity after 3000 cycles at 1 A g^−1^.

## 2 Results and Discussion

**Figure 1:**
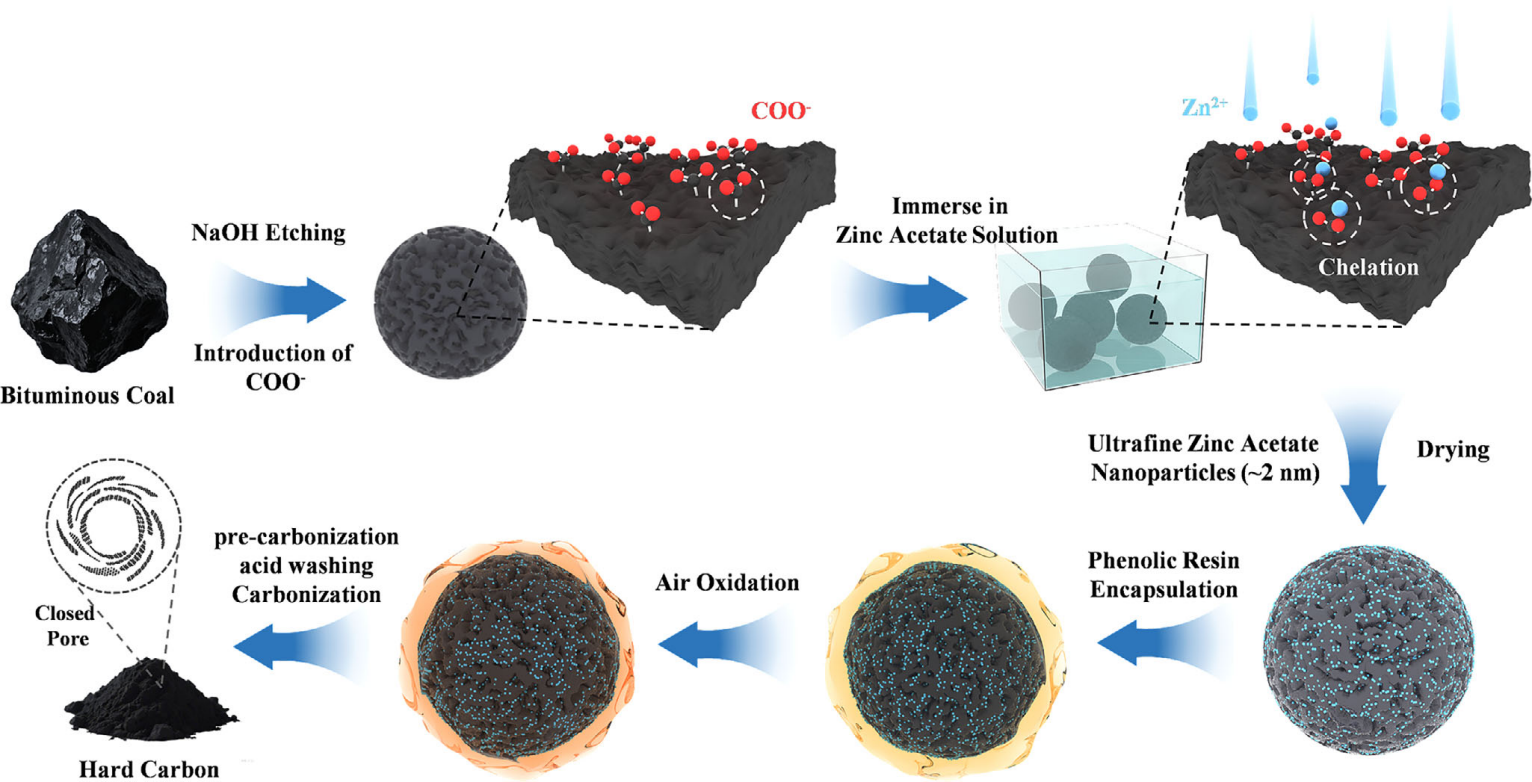
Schematic diagram of HC material preparation.

**Figure** **1** illustrates the schematic representation of the preparation procedure for coal‐based HC. Initially, bituminous coal (BC) is co‐heated with NaOH solution, yielding alkali‐etched bituminous coal (ABC) that exhibits an increased specific surface area and abundant carboxyl functional groups. Subsequently, ABC is immersed in a ZA aqueous solution and dried. The strong chelation between carboxyl groups and Zn^2+^ facilitates the uniform dispersion and precise size control of zinc acetate nanoparticles (diameter ≈2 nm).^[^ ^28^, ^29^ ^]^ Next, the sample is immersed in an ethanol solution of PR. Upon drying, the PR encapsulates the templates (ABC‐PR‐ZA). Through pre‐oxidation treatment (150 °C), the cross‐linking fixation of phenolic resin molecules is enhanced (ABC‐PR‐ZAX). Thereafter, the material undergoes pre‐carbonization (700 °C), acid washing to remove the zinc‐based templates, and finally high‐temperature carbonization (1400 °C), resulting in HC with a closed‐pore‐rich structure.

**Figure 2:**
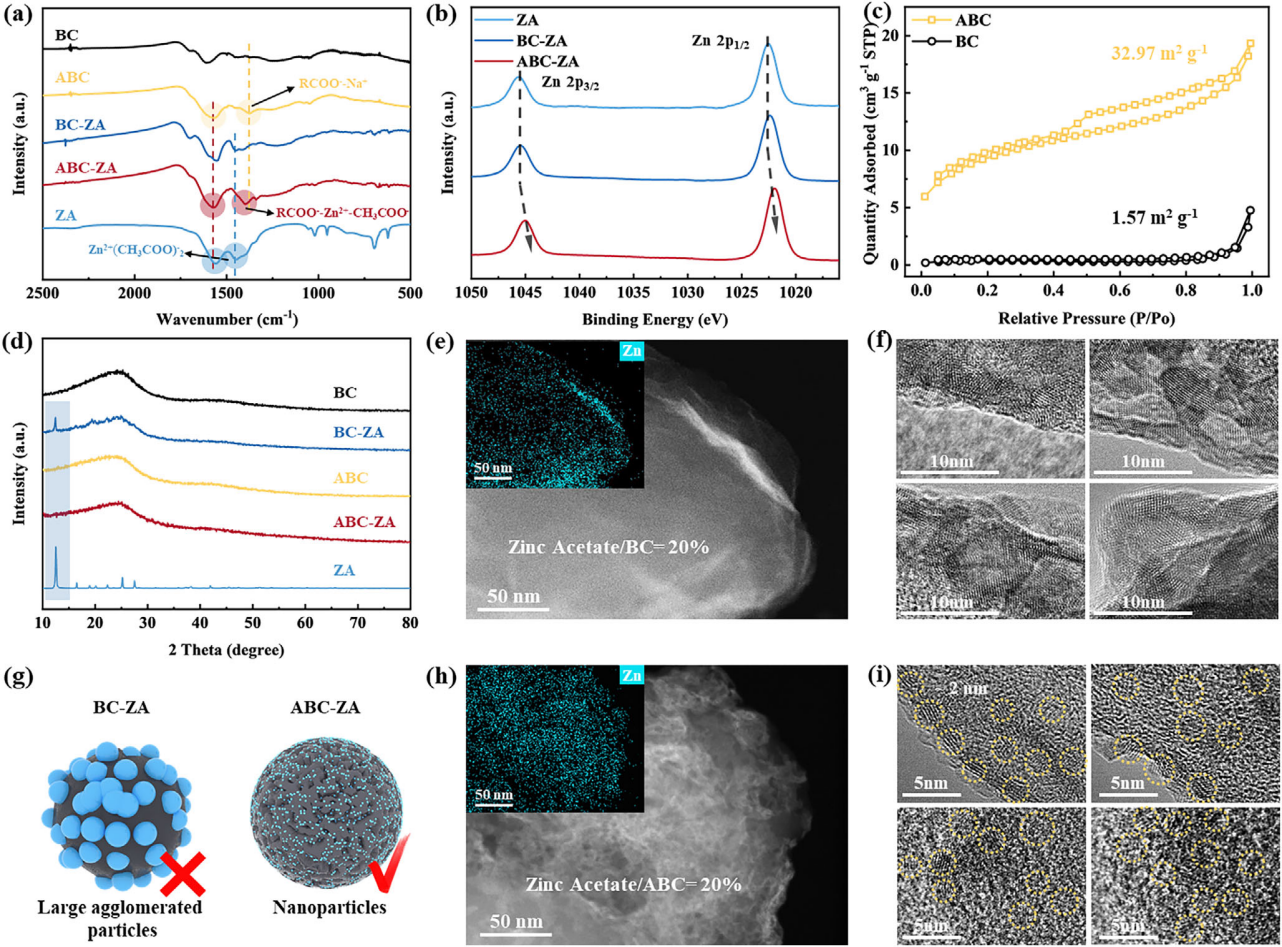
a) The FTIR spectra of BC, ABC, BC‐ZA, ABC‐ZA, ZA. b) High‐Resolution Zn 2p XPS Spectra of ZA, BC‐ZA, and ABC‐ZA. c) Nitrogen Isothermal Adsorption/Desorption Curves for ABC and BC. d) XRD patterns of the samples. e) TEM Image and EDS elemental map of BC‐ZA. f) HRTEM images of ZA particles loaded on BC. g) Schematic representation of ZA deposition on BC and ABC substrates. h) TEM Image and EDS elemental map of ABC‐ZA. i) HRTEM images of ZA particles loaded on ABC.

**Figure** **2**a presents the Fourier‐Transform Infrared (FTIR) spectra of various precursors. After NaOH treatment, ABC exhibits asymmetric absorption peaks of carboxylates at 1565 and 1375 cm^−1^.^[^ ^30^, ^31^ ^]^ the X‐ray photoelectron spectroscopy  (XPS) analysis reveals that, in comparison to BC, the concentrations of C═O and O─C═O functional groups in ABC exhibit a significant increase (Figure S3, Supporting Information), confirming the successful introduction of abundant carboxyl functional groups on the coal surface. To investigate the chelation between ZA and carboxyl groups on the ABC surface, FTIR measurements are performed on BC loaded with 20 wt% ZA (BC‐ZA), ABC loaded with 20 wt% ZA (ABC‐ZA), and pure ZA. Compared to ABC, the carboxyl‐related absorption peaks in ABC‐ZA shift to higher wavenumbers (from 1569 to 1574 cm^−1^ and from 1375 to 1402 cm^−1^). In contrast to ZA, the absorption peak at 1454 cm^−1^ disappears in ABC‐ZA, and the peak at 1556 cm^−1^ shifts to 1574 cm^−1^.^[^ ^32^, ^33^ ^]^ Notably, the absorption peaks of BC‐ZA remain nearly identical to those of ZA. Furthermore, XPS Zn 2p spectral analysis demonstrates that the binding energies of Zn 2p_3/2_ and Zn 2p_1/2_ levels for ZA and BC‐ZA remain largely unchanged, whereas those for ABC‐ZA significantly shift to lower binding energies (Figure 2b). The FTIR and XPS analysis results collectively demonstrate that the chemical environment of ZA on the ABC surface has experienced substantial alterations, thereby further substantiating the chelation interaction between ZA and surface carboxyl groups in ABC.^[^ ^34^ ^]^ By contrast, BC‐ZA merely displays characteristics of physical adsorption.

The Brunauer–Emmett–Teller (BET) analysis reveals that the BC exhibits an almost pore‐free surface, with a specific surface area of merely 1.57 m^2^ g^−1^. NaOH etching treatment leads to the development of numerous open pores (30–40 nm in diameter) on the ABC surface, resulting in a significant increase in specific surface area to 32.97 m^2^ g^−1^ (Figure 2c; Figure S2, Supporting Information).^[^ ^35^ ^]^ The XRD patterns indicate that BC‐ZA displays well‐defined crystalline peaks consistent with the ZA phase (2θ = 12.5°), whereas ABC‐ZA, similar to BC and ABC, exhibits only a broadened diffraction peak centered at ≈25° (Figure 2d). This loss of crystallinity can be ascribed to the nanoscale reduction in ZA particle size, which results in the attenuation of characteristic diffraction peak intensities and the emergence of amorphous features. The transmission electron microscopy (TEM) observation results are highly consistent with the XRD analysis results. ZA aggregates are distinctly observed on the surface of BC‐ZA (Figure 2e,f), whereas ABC‐ZA achieves uniform anchoring of ≈2 nm ZA particles via carboxyl group coordination and high specific surface area effects (Figure 2 h,i; Figure S7, Supporting Information). The highly dispersed nanoscale ZA particles on ABC‐ZA serve as precise templates for constructing HC's closed‐pore structure.

TEM and EDS analyses of ABC composites with ZA loadings of 10, 20, and 30 wt.% (Figure S5, Supporting Information) demonstrate loading‐dependent morphological evolution. At 10 wt.%, sparse zinc signals suggest incomplete surface coverage. The 20 wt% loading achieves optimal zinc distribution matching ABC morphology, whereas 30 wt.% induces nanoparticle agglomeration (evidenced by EDS zinc hotspots). The ABC‐ZA with a ZA loading of 20 wt.% is selected, where nanoparticles are effectively immobilized via a phenolic resin encapsulation layer (Figure S4, Supporting Information), named ABC‐PR‐ZA(20%), followed by air pre‐oxidation (150 °C), designated as ABC‐PR‐ZAX(20%). Subsequently, pre‐carbonization is conducted. XPS and FTIR analyses during the carbonization process at various temperatures reveal that ZA is oxidized to zinc oxide during the pre‐carbonization stage (Figure S8, Supporting Information), while both ABC and PR undergo preliminary carbonization. Acid washing removes zinc oxide, generating nanoscale pores that subsequently develop into closed pores through graphite layer reorganization during high‐temperature carbonization (1400 °C). Complete zinc removal in the final HC is confirmed by XPS (Figure S10, Supporting Information).

**Figure 3:**
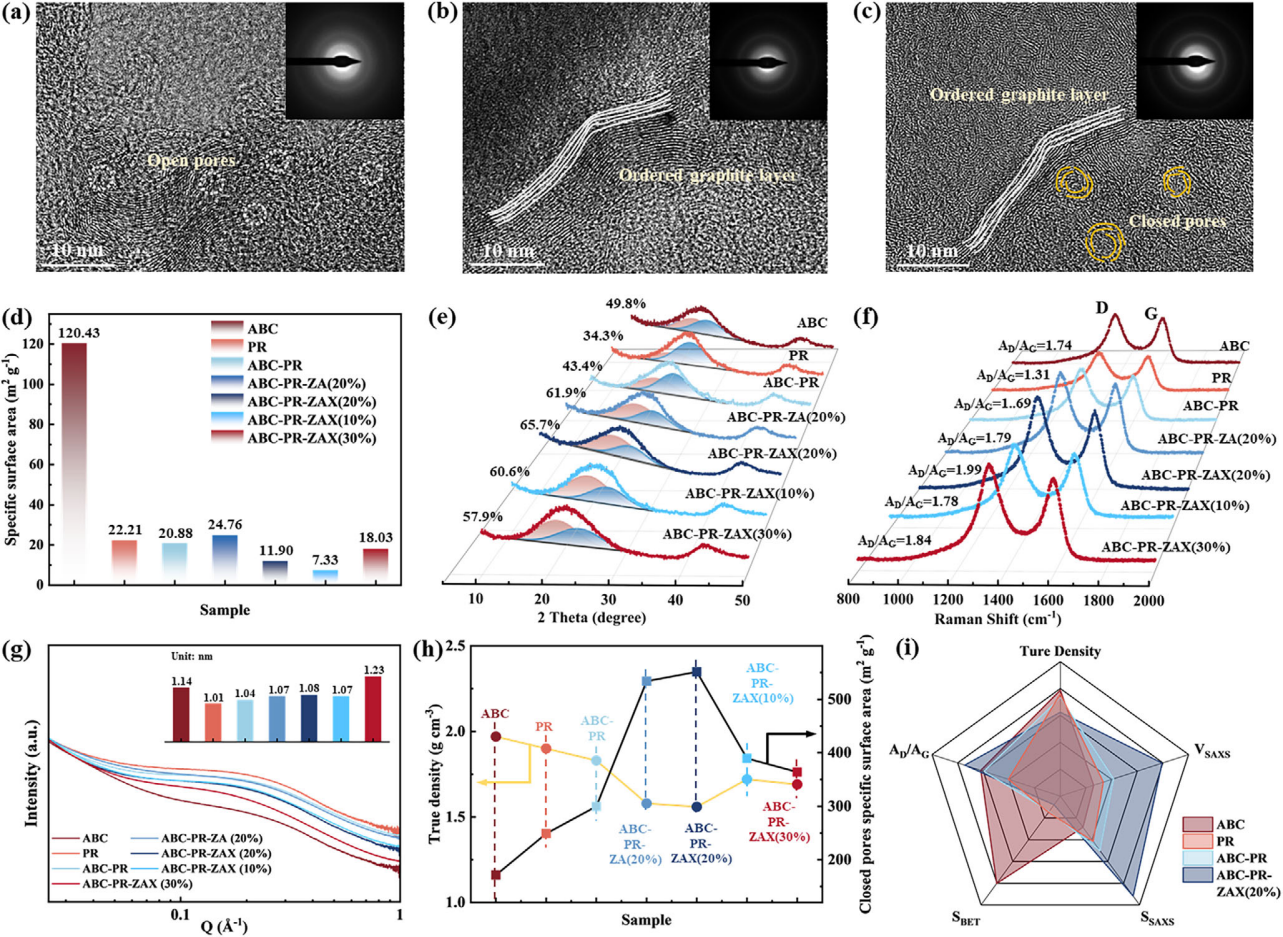
a–c) HRTEM images of HCs derived from ABC, PR, and ABC‐PR‐ZAX(20%). d) Specific surface area of HCs. e) XRD patterns and fitting curves of HCs. f) Raman spectra of HCs. g) The SAXS curves of HCs and corresponding closed‐pore radii. h) True density and closed‐pore specific surface area in HCs. i) Radar chart comparing structural parameters among different HCs.

HRTEM characterization elucidates the structural features of HCs derived from ABC, PR, and ABC‐PR‐ZAX (20%) after 1400 °C high‐temperature carbonization. The HC derived from ABC shows abundant open pores (**Figure** **3**a), contrasting with PR‐derived HC's ordered graphite microcrystals (Figure 3b). Owing to PR encapsulation, the HC derived from ABC‐PR‐ZAX(20%) demonstrates a uniform and well‐ordered graphite coating post‐carbonization, with a thickness of ≈4 nm, and contains numerous closed pores within the encapsulated graphite layer region (Figure 3c). Notably, no large‐scale open pore structures akin to those in Figure 3a are observed, suggesting that PR not only effectively encapsulates ZA nanoparticles to form closed pores but also significantly suppresses the formation of open pores during co‐carbonization by covering the ABC surface, thereby contributing to enhanced ICE of the HC (Figure 3c).

The specific surface area of the HCs obtained after carbonization at 1400 °C for each sample is systematically characterized using N_2_ adsorption–desorption isotherm (Figure 3d; Figure S12, Supporting Information). Among these, the HC derived from ABC exhibits a high specific surface area of 120.43 m^2^ g^−1^ due to the presence of numerous open pores on its surface. In contrast, the HC derived from PR has a significantly lower specific surface area of 22.21 m^2^ g^−1^. After PR coating, the specific surface area of the HC derived from ABC‐PR (ABC:PR = 1:0.8 wt) decreases to 20.88 m^2^ g^−1^, indicating that PR coating effectively suppresses the formation of open pores. Furthermore, the HC derived from ABC‐PR‐ZA(20%) exhibits a specific surface area of 24.76 m^2^ g^−1^, whereas the HC derived from ABC‐PR‐ZAX(20%), following air pre‐oxidation cross‐linking treatment, shows a reduced specific surface area of 11.90 m^2^ g^−1^. This reduction is attributed to air pre‐oxidation promoting precursor cross‐linking, thereby significantly inhibiting pore formation.^[^ ^36^ ^]^ To investigate the influence of different ZA loading amounts on the structure of the HCs, the specific surface areas of the precursor‐derived HCs with ZA loadings of 10 and 30 wt.% are measured. The results reveal that the HC derived from ABC‐PR‐ZAX(10%) has a specific surface area of 7.33 m^2^ g^−1^, while the HC derived from ABC‐PR‐ZAX(30%) exhibits a higher specific surface area of 18.03 m^2^ g^−1^. This phenomenon may be associated with incomplete PR encapsulation caused by excessive ZA loading.

The influence of different precursor structures and ZA loading amounts on the structural characteristics of the HCs is systematically investigated using XRD and Raman spectroscopy techniques (Figure 3e,f).^[^ ^37^, ^38^ ^]^ As depicted in the XRD pattern in Figure 3e, two broad diffraction peaks are observed at 24° and 43°, corresponding to the (002) and (100) crystal planes, respectively. Notably, the asymmetry of the (002) peak indicates the coexistence of ordered and disordered graphite‐like phases within the HC, a feature that can be further quantified through fitting analysis.^[^ ^39^ ^]^ As shown in Figure 3f and Figure S11 (Supporting Information), the Raman spectrum is decomposed into four Lorentzian peaks, corresponding to the D peak (1345 cm^−1^), D_2_ peak (1200 cm^−1^), D_3_ peak (1500 cm^−1^), and G peak (1590 cm^−1^).^[^ ^40^, ^41^ ^]^ Among these, the ratio of the D peak area to the G peak area (A_D_/A_G_) serves as an indicator for quantitatively assessing the degree of structural disorder, with higher values reflecting increased amorphization. In the HCs derived from ABC and PR, the amorphous carbon content is 49.8% and 34.3%, respectively, with corresponding A_D_/A_G_ ratios of 1.74 and 1.31. These findings align with the microstructural features observed in the TEM images presented in Figure 3b, confirming the higher graphitization degree of the HC derived from PR. The disorder level of the HC derived from ABC‐PR lies between those of ABC and PR (A_D_/A_G_ = 1.69, amorphous carbon content 43.4%). The introduction of the ZA templates significantly alters the microstructure of the material. Under a 20 wt.% ZA loading condition (relative to the mass of ABC), the A_D_/A_G_ value of the HC derived from ABC‐PR‐ZA(20%) increases to 1.79, primarily due to the spatial hindrance effect induced by nanoparticles, which inhibits the stacking of graphite layers and enhances the amorphous carbon content to 61.9%. Furthermore, pre‐oxidation treatment further intensifies the disorder, resulting in an A_D_/A_G_ value of 1.99 for the HC derived from ABC‐PR‐ZAX(20%). This enhancement is attributed to the covalent cross‐linking of the organic precursor, which suppresses the kinetics of graphitization and leads to the formation of a highly defective carbon matrix with an amorphous carbon content of 65.7%.^[^ ^42^ ^]^ When the ZA loading is reduced to 10% of the ABC weight, incomplete nanoparticle coverage weakens the spatial hindrance effect, decreasing the amorphous carbon content to 60.6% (A_D_/A_G_ = 1.78). Conversely, at a ZA loading of 30% of the ABC weight, excessive aggregation of ZA particles on the ABC surface diminishes the spatial hindrance effect, leading to an amorphous carbon content of 57.9% and an A_D_/A_G_ value of 1.84.

The closed‐pore structures of the HCs derived from various precursors are systematically analyzed by small‐angle X‐ray scattering (SAXS) and true density tests (Figure 3g,h; Table S2, Supporting Information).^[^ ^43^ ^]^ Ideal graphite is considered to have no closed pores, with a true density of 2.26 g cm^−3^. Therefore, the lower the true density, the larger the volume of closed pores in the carbon material, and the more space available for sodium ion filling. All SAXS curves exhibit a relatively broad peak at 0.1 A^−1^, indicating that, except for the HCs prepared through the ZA templates, there is also a certain number of closed pores in the HCs derived from ABC and PR, but their content is relatively low. Specifically, the closed‐pore radii of the HCs derived from ABC and PR are 1.14 and 1.01 nm, respectively (Figure 3g), and the corresponding true densities are 1.97 and 1.90 g cm^−3^, respectively. To facilitate the analysis of the pore structure, it is usually assumed that the closed pores are approximately spherical, and the specific surface area of the closed pores is calculated based on the closed‐pore radius. Thus, the specific surface areas of the closed pores of the HCs derived from ABC and PR are 171.41 and 249.02 cm^2^ g^−1^, respectively. The true density of the HC derived from ABC‐PR decreases to 1.83 g cm^−3^, and the specific surface area of the closed pores increases to 299.91 cm^2^ g^−1^, with a closed‐pore radius of 1.04 nm. The reduced true density indicates that the PR coating has a certain pore‐forming effect, but the effect is limited. Under the action of the nano ZA particle templates, the true density of the HC derived from ABC‐PR‐ZA(20%) significantly decreases to 1.58 g cm^−3^, and the specific surface area of the closed pores increases to 533.92 cm^2^ g^−1^, with a closed‐pore radius of 1.07 nm. After air pre‐oxidation treatment, the true density of the HC derived from ABC‐PR‐ZAX(20%) is 1.56 g cm^−3^, and the specific surface area of the closed pores reaches 551.52 cm^2^ g^−1^, with a closed‐pore radius of 1.08 nm, indicating that air pre‐oxidation has a minor effect on the closed‐pore structure. For the HC derived from ABC‐PR‐ZAX(10%), due to incomplete coverage of the nano ZA particles, the true density is 1.72 g cm^−3^, the specific surface area of the closed pores is 389.48 cm^2^ g^−1^, and the closed‐pore radius is 1.07 nm. For the HC derived from ABC‐PR‐ZAX(30%), due to excessive accumulation of nanoparticles, the pore‐forming effect weakens and the closed‐pore radius increases to 1.23 nm, with a true density of 1.69 g cm^−3^ and a specific surface area of the closed pores of 363.99 cm^2^ g^−1^. Based on the results of various characterization techniques, the HC derived from ABC‐PR‐ZAX(20%) exhibits the highest carbon layer disorder, the lowest specific surface area, the lowest true density, and the largest specific surface area of the closed pores, with a moderate closed‐pore diameter, making it highly suitable as a negative electrode material for SIBs (Figure 3i).

**Figure 4:**
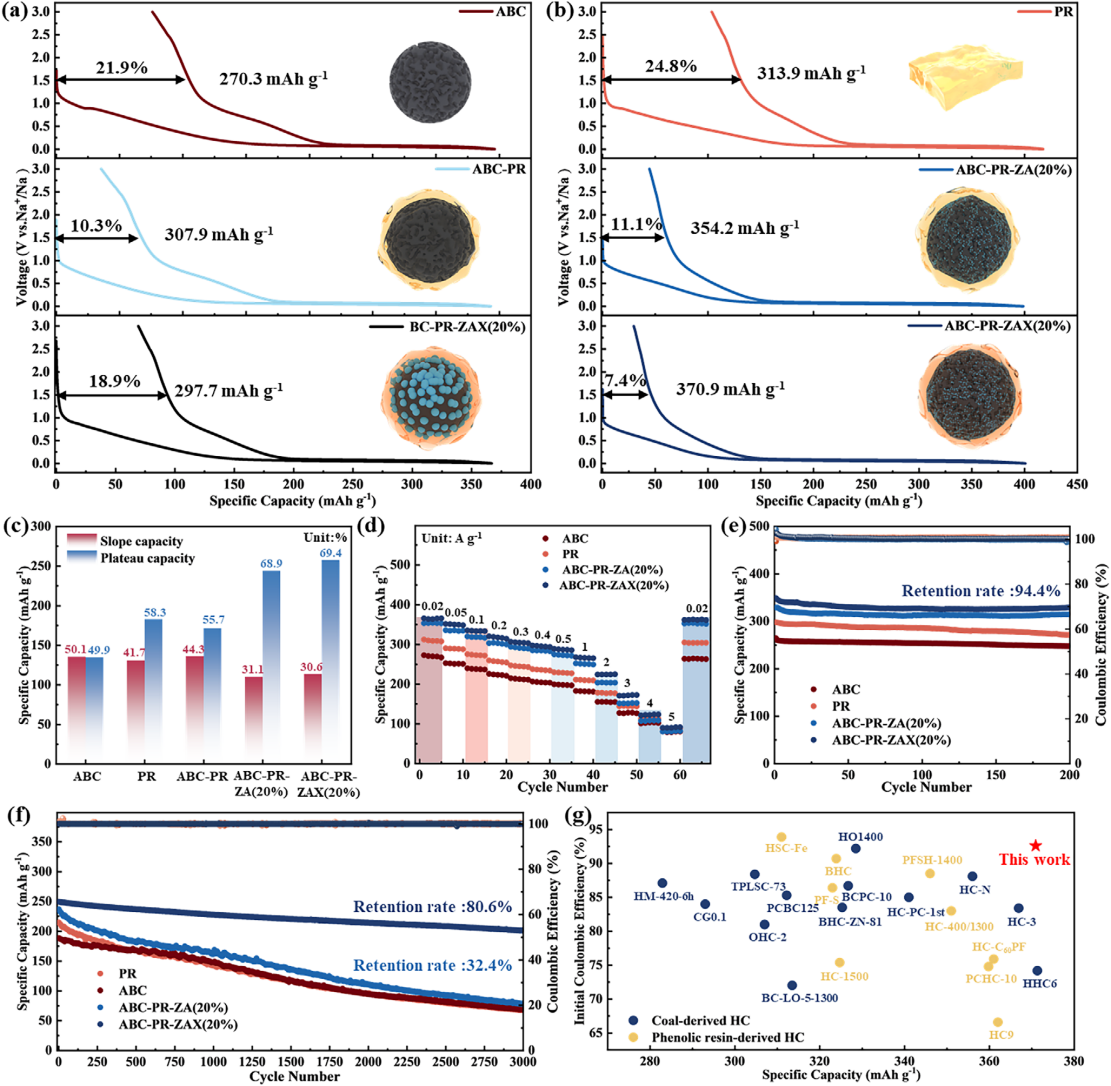
a,b) The first‐cycle charge–discharge curves of HCs derived from ABC, PR, ABC‐PR, ABC‐PR‐ZA, BC‐PR‐ZAX(20%), and ABC‐PR‐ZAX(20%). c) Comparison of plateau and slope capacities among the HCs. d) Rate performance of the HCs. e) Cycling performance of the HCs at 100 mA g^−1^. f) Cycling performance of the HCs at 1000 mA g^−1^. g) A comparison of the specific capacity and ICE of the ABC‐PR‐ZAX(20%) derived HC with those of other reported HC materials.

Electrochemical evaluations elucidate the critical structure‐performance relationships in the developed HC anodes. The HC derived from ABC exhibits limited sodium storage capability, with a reversible capacity of 270.3 mAh g^−1^ and an ICE of 78.0%. This limitation is primarily attributed to its predominantly open‐pore structure, where the platform capacity contributes only 49.9% of the total storage capacity (**Figure** **4**a,b). The HC derived from PR demonstrates a specific capacity of 313.9 mAh g^−1^ and an ICE of 75.2%. Notably, after PR coating, the HC derived from ABC‐PR achieves a significantly enhanced ICE of 89.44%, while maintaining a specific capacity of 307.72 mAh g^−1^. Furthermore, the incorporation of ZA templates in ABC‐PR‐ZA(20%) results in a more abundant closed‐pore structure, which facilitates sodium ion storage. The HC derived from ABC‐PR‐ZA(20%) achieves a reversible specific capacity of 354.24 mAh g^−1^, an ICE of 88.8%, and increases the platform capacity contribution to 68.9% (Figure 4c). Pre‐oxidation treatment further optimizes the graphitization structure, enabling the HC derived from ABC‐PR‐ZAX(20%) to exhibit superior electrochemical performance, including a high reversible specific capacity of 370.96 mAh g^−1^, an ICE of 92.5%, a platform capacity of 69.4%, and excellent rate capability (Figure 4b,d). To validate the effectiveness of carboxyl chelation‐induced nano ZA templates formation for constructing closed‐pore structures, BC without NaOH etching substitutes for ABC under otherwise identical material ratios and processing conditions to prepare the HC derived from BC‐PR‐ZAX(20%). Experimental results indicate that this material exhibits a specific capacity of only 297.7 mAh g^−1^ and an ICE of 81.1%, markedly inferior to the performance of ABC‐PR‐ZAX(20%) (Figure 4a). The regulation of ZA loading has a significant impact on the material performance: when the loading is 10% and 15%, due to insufficient template coverage, the lower loading leads to a sparse distribution of ZA nanoparticles on the ABC surface, resulting in a limited number of closed pores and difficulty in generating sufficient steric hindrance effect to effectively inhibit the ordered stacking of graphite layers, thus the electrochemical performance is poor (Figure S14, Supporting Information). When the loading increases to 25% and 30%, the excessive ZA content causes particle agglomeration on the ABC surface, reducing the dispersion and effectiveness of the template, which also adversely affects the electrochemical performance (Figure S14, Supporting Information).

At a current density of 100 mA g^−1^, the HC derived from ABC‐PR‐ZAX(20%) exhibits a capacity retention of 94.4% after 200 cycles (Figure 4e). At 1000 mA g^−1^, after 3000 cycles, the HC derived from ABC‐PR‐ZAX(20%) demonstrates a capacity retention of 80.4%, whereas the HC derived from ABC‐PR‐ZA(20%) without pre‐oxidation treatment achieves only 32.4% (Figure 4f). This substantial difference can be ascribed to the pre‐oxidation treatment, which enhances the cross‐linking degree of the precursor, reduces the specific surface area of the HC, and improves structural stability, thereby significantly boosting high‐rate cycling performance. Figure 4g presents a comparison of the specific capacity and ICE between recently reported coal‐based HCs and phenolic resin‐based HCs. The results indicate that the HC derived from ABC‐PR‐ZAX(20%) in this work exhibits superior performance in both specific capacity and ICE.^[^ ^18^, ^23^, ^25^, ^44^, ^45^, ^46^, ^47^, ^48^, ^49^, ^50^, ^51^, ^52^, ^53^, ^54^, ^55^, ^56^, ^57^, ^58^, ^59^, ^60^, ^61^ ^]^

**Figure 5:**
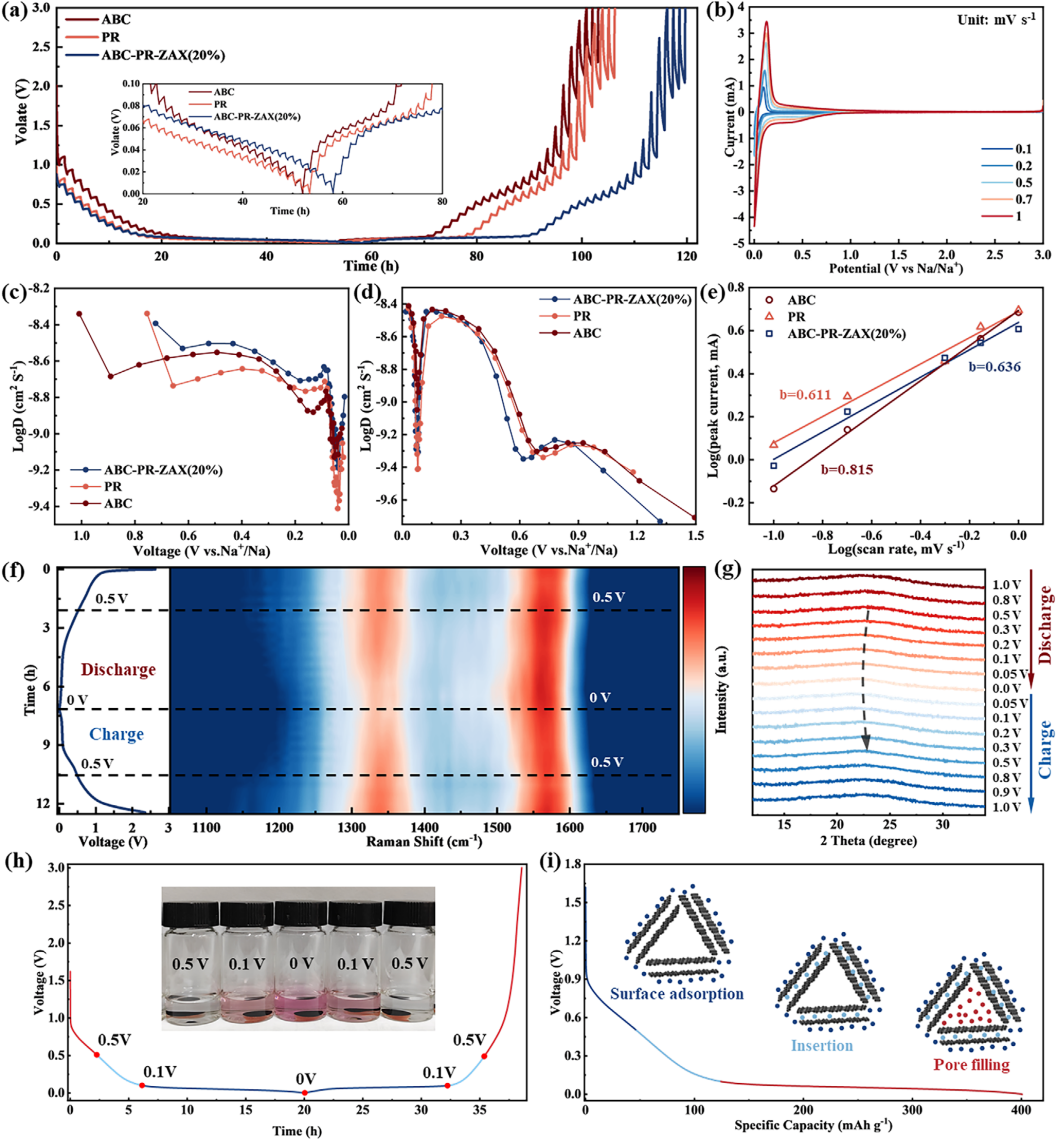
a) GITT curves of HCs derived from ABC, PR, and ABC‐PR‐ZAX(20%). b) Cyclic voltammogram of the HC derived from ABC‐PR‐ZAX(20%) at different scan rates. c,d) Sodium diffusion coefficients in HCs during discharge and charge processes. e) *b* value of HCs calculated from cyclic voltammograms at different scan rates. f) In situ Raman spectra of the HC derived from ABC‐PR‐ZAX(20%). g) In situ XRD spectra of the HC derived from ABC‐PR‐ZAX(20%). h) The color variations of the phenolphthalein ethanol solution following its reaction with the HC derived from ABC‐PR‐ZAX(20%) electrode under varying voltage conditions. i) Sodium storage schematic of the HC derived from ABC‐PR‐ZAX(20%).

The sodium ion diffusion rate (D_Na+_) is quantitatively analyzed via the galvanostatic intermittent titration technique (GITT, **Figure** **5**a) to elucidate the transmission kinetics characteristics at different stages. Prior to discharging to 0.1 V, all samples demonstrate a relatively high sodium ion diffusion coefficient (Figure 5c,d), which is strongly associated with the adsorption behavior of sodium ions on the material surface. However, within the voltage range of 0.1–0 V, the sodium ion diffusion coefficient decreases markedly as the discharge depth increases, indicating that the diffusion kinetics during sodium ion intercalation into the graphite layer and pore filling remain relatively sluggish.^[^ ^62^, ^63^ ^]^ The sodium storage behavior of HCs is further explored through cyclic voltammetry (CV) curves obtained at scan rates ranging from 0.1 to 1 mV s^−1^ (Figure 5b,e; Figure S15, Supporting Information). The b value is calculated to characterize the diffusion behavior of sodium ions in HCs and to assess the capacitance contribution rate at various scan rates.^[^ ^44^ ^]^ The HC derived from ABC exhibits a relatively high b value (0.816), with a capacitance contribution rate of 72% at a scan rate of 0.1 mV s^−1^ (Figure S16, Supporting Information), which is closely linked to its large specific surface area. In contrast, the HC derived from PR has a b value of 0.611 and a capacitance contribution rate of 54%. Meanwhile, the HC derived from ABC‐PR‐ZAX(20%) after closed‐pore engineering exhibits a b value of 0.631, with the capacitance contribution rate decreasing to 48% under the same conditions, representing the lowest value across all scan rates. This indicates that the sodium storage mechanism of this material is predominantly governed by diffusion control.

Combined with in situ Raman spectroscopy analysis (Figure 5f), it is observed that for the HC derived from ABC‐PR‐ZAX(20%), both the D band and G band shift to lower frequencies upon discharging below 0.5 V, accompanied by a decrease in the absorption intensity of the D band. This indicates that sodium ions are initially adsorbed at active sites on the material surface (above 0.5 V). Subsequently, the G band continues to red‐shift while the D band remains relatively stable, suggesting that sodium ions insert into the HC layers, thereby weakening the C─C bond strength.^[^ ^64^ ^]^ Upon charging, the positions of the D and G band revert to their initial states, confirming the excellent reversibility of this process. In the in situ XRD spectra (Figure 5g), the 002 peak located at 23°–24° exhibits periodic changes throughout the charge‐discharge cycle. During discharge, the insertion of sodium ions results in an increase in the interlayer spacing of the carbon layers, causing the 002 peak to shift to a lower angle. During charging, as sodium ions are extracted, the material gradually returns to its initial state. The phenolphthalein impregnation test (Figure 5h) directly validates the pore‐filling mechanism. The electrode discharged to 0.5 V does not induce any color change, indicating that sodium ions are predominantly adsorbed on the surface at this stage. The electrode discharged to 0.1 V causes the solution to turn light purple, indicating the onset of sodium ion filling in closed pores and the formation of sodium clusters.^[^ ^10^, ^65^ ^]^ The electrode discharged to 0 V induces a strong purple–red coloration (Figure S17, Supporting Information), indicating the formation of more quasi‐metallic sodium within the closed pores at this stage. These multimodal analyses collectively confirm that the HC derived from ABC‐PR‐ZAX(20%) exhibits a three‐stage sodium ion storage mechanism, including surface adsorption above 0.5 V, interlayer insertion between 0.5 and 0.1 V, and pore filling within closed pores between 0.1 and 0 V.

**Figure 6:**
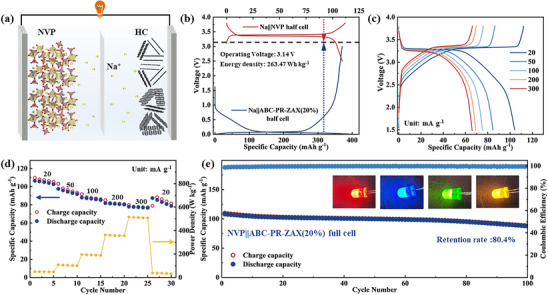
a) Schematic of SIBs with HC anode and NVP cathode. b) The first‐cycle charge‐discharge curves of the Na3V2(PO4)3 cathode and the anode of the HC derived from ABC‐PR‐ZAX(20%) at 20 mA g^−^1. c) Rate capability of Na3V2(PO4)3//ABC‐PR‐ZAX(20%) full cell. d) The rate performance and power density of the Na3V2(PO4)3//ABC‐PR‐ZAX(20%) full cell. e) Cyclability of the Na3V2(PO4)3//ABC‐PR‐ZAX(20%) full cell at 1C, the insert shows the optical photographs of the lighted LED bulbs.

To evaluate the practical application potential of the HC derived from ABC‐PR‐ZAX(20%), a sodium‐ion full cell (denoted as NVP//ABC‐PR‐ZAX(20%)) is assembled, employing a commercial Na_3_V_2_(PO_4_)_3_ (NVP) cathode and the HC anode derived from ABC‐PR‐ZAX(20%), as illustrated in **Figure** **6**a. The charge–discharge curves of the NVP cathode and the HC‐derived anode from ABC‐PR‐ZAX(20%) are depicted in Figure 6b. At a current density of 20 mA g^−1^, the NVP//ABC‐PR‐ZAX(20%) full cell exhibits a high sodium storage capacity of 104.13 mAh g^−1^ and an energy density of 263.47 Wh kg^−1^, with an operating voltage of ≈3.14 V. Figure 6c,d respectively present the charge–discharge curves, specific capacities, and power densities of the full cell at various current densities. Specifically, at current densities of 20, 50, 100, 200, and 300 mA g^−1^, the full cell maintains capacities of 104.13, 84.62, 75.18, 69.23, and 65.95 mAh g^−1^, respectively. Furthermore, after 100 cycles at a current density of 100 mA g^−1^, the capacity retention rate reaches 80.4%. These findings demonstrate that the HC anode material derived from ABC‐PR‐ZAX(20%) holds significant promise for practical applications in future SIBs.

## 3 Conclusion

This work introduces a carboxyl‑guided, ultrafine ZA templating strategy that—together with phenolic‑resin encapsulation—enables deterministic closed‑pore engineering in coal‑derived HC. As a negative electrode for SIBs, it demonstrates a reversible capacity of 370.96 mAh g^−1^, an ICE of 92.5%, and a capacity retention rate of 80.4% after 3000 cycles at 1 A g^−1^. The “chelation‐encapsulation‐interfacial pore formation” synergistic mechanism proposed in this work establishes a new paradigm for the pore‐closing engineering of HC anodes.

## Conflict of Interest

The authors declare no conflict of interest.

## Supporting information



Supporting Information

## Data Availability

The data that support the findings of this study are available from the corresponding author upon reasonable request.

## References

[1] H. He , J. He , H. Yu , L. Zeng , D. Luo , C. Zhang , Adv. Energy Mater. 2023, 13, 2300357.

[2] X. Chen , P. Liu , T. Wang , C. He , W. Song , J. Ban , S. Gao , K. Liu , J. Mater. Chem. A 2025, 13, 20650.

[3] X. Fan , X. Kong , P. Zhang , J. Wang , Energy Storage Mater. 2024, 69, 103386.

[4] R. Ma , Y. Chen , Q. Li , B. Zhang , F. Chen , C. Leng , D. Jia , N. Guo , L. Wang , Chem. Eng. J. 2024, 493, 152389.

[5] L. Wang , Z. Xu , P. Lin , Y. Zhong , X. Wang , Y. Yuan , J. Tu , Adv. Energy Mater. 2025, 15, 2403084.

[6] N. LeGe , X.‐X. He , Y.‐X. Wang , Y. Lei , Y.‐X. Yang , J.‐T. Xu , M. Liu , X. Wu , W.‐H. Lai , S.‐L. Chou , Energy Environ. Sci. 2023, 16, 5688.

[7] Q. Xia , C.‐L. Ko , E. R. Cooper , Q. Gu , R. Knibbe , J. R. Harmer , Adv. Funct. Mater. 2025, 35, 2421976.

[8] H. Zhang , S. Lin , C. Shu , Z. Tang , X. Wang , Y. Wu , W. Tang , Mater. Today 2025, 85, 231.

[9] J. C. Hyun , H. M. Jin , J. H. Kwak , S. Ha , D. H. Kang , H. S. Kim , S. Kim , M. Park , C. Y. Kim , J. Yoon , J. S. Park , J.‐Y. Kim , H.‐D. Lim , S. Y. Cho , H.‐J. Jin , Y. S. Yun , Energy Environ. Sci. 2024, 17, 2856.

[10] X. Ji , Y. Wei , H. Yang , Z. Lu , S. Jin , H. Jin , X. Kong , H. Ji , Small 2024, 20, 2402616. 10.1002/smll.202402616 39031846

[11] L. Xu , Y. Li , Y. Xiang , C. Li , H. Zhu , C. Li , G. Zou , H. Hou , X. Ji , ACS Nano 2025, 19, 14627. 40259809 10.1021/acsnano.5c02665

[12] R. Zhou , S. Peng , Z. Wang , Y. Zhao , C. Bao , Y. Xia , Y. Zhu , H. Yang , Z. Bo , Q. Yu , Adv. Funct. Mater. 2025, 35, 2423530.

[13] S. Ma , W. Yan , Y. Dong , Y. Su , L. Ma , Y. Li , Y. Fang , B. Wang , S. Wu , C. Liu , S. Chen , L. Chen , Q. Huang , J. Wang , N. Li , F. Wu , Mater. Today 2024, 75, 334.

[14] Q. Zhang , P. Yue , M. Jia , J. Jia , Y. Ren , G. Li , J. Sun , L. Hou , M. Chen , C. Yuan , Chem. Eng. J. 2024, 500, 156779.

[15] L. Kitsu Iglesias , E. N. Antonio , T. D. Martinez , L. Zhang , Z. Zhuo , S. J. Weigand , J. Guo , M. F. Toney , Adv. Energy Mater. 2023, 13, 2302171.

[16] W. Li , J. Li , B. W. Biney , Y. Yan , X. Lu , H. Li , H. Liu , W. Xia , D. Liu , K. Chen , A. Guo , Energy Storage Mater. 2025, 74, 103867.

[17] Y. Zhang , G. Wang , P. Yue , J. Sun , M. Gao , J. Wang , L. Hou , M. Chen , C. Yuan , Adv. Funct. Mater. 2025, 35, 2414761.

[18] Z. Zhou , Z. Wang , Y. Zhang , Q. Lin , Q. Jing , S. Yan , J. Guo , Y. Shuai , L. Fan , Composites, Part B 2025, 303, 112562.

[19] X.‐Y. Wang , K.‐Y. Zhang , M.‐Y. Su , H.‐H. Liu , Z.‐Y. Gu , D. Dai , B. Li , J.‐W. Wang , X.‐Y. He , X.‐L. Wu , Carbon 2024, 229, 119526.

[20] K. Liu , S. Ji , B. Liu , X. Zeng , Y. Zhang , X. Wu , X. Zhou , J. Xiao , L. Li , Y. Zhang , Nano Res. 2025, 18, 94907625.

[21] Y. Li , Y.‐S. Hu , X. Qi , X. Rong , H. Li , X. Huang , L. Chen , Energy Storage Mater. 2016, 5, 191.

[22] G. Liu , J. Yuan , H. Li , Z. Li , C. Hu , X. Qiao , M. Wang , B. Yuan , P. Zhang , Z. Wu , ACS Appl. Mater. Interfaces 2024, 16, 46226. 39172642 10.1021/acsami.4c07654

[23] G. Liu , J. Yuan , Z. Li , H. Li , C. Wang , Z. Zeng , C. Hu , J. Yang , B. Yuan , J. Zhang , Z. Wu , Carbon 2025, 235, 120085.

[24] M.‐Y. Su , K.‐Y. Zhang , E. H. Ang , X.‐L. Zhang , Y.‐N. Liu , J.‐L. Yang , Z.‐Y. Gu , F. A. Butt , X.‐L. Wu , Rare Met. 2024, 43, 2585.

[25] W. Fu , G. Zhao , S. He , C. Yan , S. Li , A. Tang , H. Yang , Small 2025, 21, 2411376. 10.1002/smll.202411376 39846289

[26] C. Wen , M. Huang , C. Feng , N. Kong , K. Hou , R. Xie , Z. Shao , R. Tan , F. Han , Carbon 2024, 230, 119702.

[27] S. Xu , J. van der Watt , D. Laudal , R. Zhang , R. Ahmed , X. Hou , J. Power Sources 2025, 628, 235858.

[28] W. Xin , J. Xiao , J. Li , L. Zhang , H. Peng , Z. Yan , Z. Zhu , Energy Storage Mater. 2023, 56, 76.

[29] B. Zhang , L. Qin , Y. Fang , Y. Chai , X. Xie , B. Lu , S. Liang , J. Zhou , Sci. Bull. 2022, 67, 955. 10.1016/j.scib.2022.01.027 36546030

[30] S. S. R. Dehkordi , Q. Delavar , H. A. Ebrahim , S. S. Partash , Mater. Today Commun. 2022, 33, 104776.

[31] Y. Zhang , R. Wang , H. Ao , T. Ma , X. Zhu , X. Zhang , J. Rong , Z. Zhou , Z. Bai , S. X. Dou , N. Wang , Z. Li , Adv. Energy Mater. 2025, 15, 2404203.

[32] J. Mirtič , J. Ilaš , J. Kristl , Carbohydr. Polym. 2018, 181, 93. 29254056 10.1016/j.carbpol.2017.10.040

[33] G. Wu , W. Yang , Y. Yang , Y.‐K. Choe , E. Yoo , ACS Nano 2025, 19, 18244. 40335072 10.1021/acsnano.4c18162

[34] K. Xie , K. Ren , C. Sun , S. Yang , M. Tong , S. Yang , Z. Liu , Q. Wang , ACS Appl. Energy Mater. 2022, 5, 4170.

[35] Q. Xu , R. Liu , H. Yang , J. Mol. Liq. 2021, 329, 115518.

[36] Z. Wei , H.‐X. Zhao , Y.‐B. Niu , S.‐Y. Zhang , Y.‐B. Wu , H.‐J. Yan , S. Xin , Y.‐X. Yin , Y.‐G. Guo , Mater. Chem. Front. 2021, 5, 3911.

[37] Y. Chu , J. Zhang , Y. Zhang , Q. Li , Y. Jia , X. Dong , J. Xiao , Y. Tao , Q.‐H. Yang , Adv. Mater. 2023, 35, 2212186. 10.1002/adma.202212186 36806260

[38] H.‐M. Xiao , H. Zheng , P. Yuan , J.‐H. Luo , L.‐L. Shen , J.‐F. Tan , X.‐Y. Luo , D. Li , Y. Chen , Rare Met. 2024, 43, 4274.

[39] Y. Liu , J. Yin , R. Wu , H. Zhang , R. Zhang , R. Huo , J. Zhao , K.‐Y. Zhang , J. Yin , X.‐L. Wu , H. Zhu , Energy Storage Mater. 2025, 75, 104008.

[40] J. Cui , W. Li , P. Su , X. Song , W. Ye , Y. Zhang , Z. Chen , Adv. Energy Mater. 2025, 15, 2502082.

[41] Z. Zheng , S. Hu , W. Yin , J. Peng , R. Wang , J. Jin , B. He , Y. Gong , H. Wang , H. J. Fan , Adv. Energy Mater. 2024, 14, 2303064.

[42] S. C. Dey , B. Worfolk , L. Lower , W. J. Sagues , M. R. Nimlos , S. S. Kelley , S. Park , ACS Energy Lett. 2024, 9, 2590.

[43] Y. Xue , Y. Chen , Y. Liang , L. Shi , R. Ma , X. Qiu , Y. Li , N. Guo , Q. Zhuang , B. Xi , Z. Ju , S. Xiong , Adv. Mater. 2025, 37, 2417886. 10.1002/adma.202417886 40275813

[44] L. Guo , C. Qiu , R. Yuan , X. Li , X. Li , K. Li , W. Zhu , X. Liu , A. Li , H. Liu , X. Chen , H. Song , ACS Appl. Mater. Interfaces 2024, 16, 27419. 38743926 10.1021/acsami.4c04101

[45] X. Li , J. Zhang , J. Zhang , L. Guo , H. Zhang , R. Yuan , H. Liu , A. Li , X. Chen , H. Song , ACS Nano 2025, 19, 14829. 40219987 10.1021/acsnano.4c18421

[46] H. Ma , Y. Tang , B. Tang , Y. Zhang , L. Deng , L. Liu , S. Dong , Y. Cao , Carbon Energy 2024, 6, 584.

[47] Y. Huang , Z. Hou , J. Wang , Y. Li , T. Ma , D. Nan , C. Wei , J.‐G. Wang , Angew. Chem., Int. Ed. 2025, 64, 202423864. 10.1002/anie.202423864 39888235

[48] Y. Chen , G. Peng , M. Zhao , Y. Zhou , Y. Zeng , H. He , J. Zeng , Carbon 2025, 238, 120269.

[49] H. Pei , R. Wang , Y. Li , Y. Xue , Y. Chen , J. Jiang , X. Kong , Q. Zhuang , Z. Ju , J. Colloid Interface Sci. 2025, 690, 137359. 40127575 10.1016/j.jcis.2025.137359

[50] Y. Lin , J. Jia , J. Wang , X. Kang , G. Huang , B. Xing , W. Kang , C. Zhang , Chem. Eng. J. 2025, 504, 158858.

[51] Y. Zhu , X. Tang , Z. Kong , Z. You , Y. Zhang , Y. Duan , Y. Zhang , Solid State Ionics 2024, 416, 116668.

[52] B. Chen , Q. Meng , T. Wang , W. Zhang , X. Qiu , J. Power Sources 2024, 624, 235566.

[53] Z. Zhou , Z. Wang , L. Fan , Chem. Eng. J. 2024, 490, 151428.

[54] L. Liu , X. Yang , N. Zhou , Chem. Eng. J. 2024, 498, 155876.

[55] B. Wang , S. Zhang , X. Jia , F. Yuan , H. Sun , Z. Li , Q. Sun , Q. Wang , D. Zhang , Chem. Eng. J. 2024, 499, 156126.

[56] L. Guo , C. Qiu , H. Liu , R. Yuan , X. Li , X. Li , K. Li , W. Zhu , M. Tabish , X. Liu , A. Li , X. Chen , H. Song , Chem. Eng. J. 2025, 512, 162478.

[57] J. Niu , J. Cheng , Z. Yi , C. Wang , X. Li , J. Chen , L. Xie , Y. Chang , X. Li , F. Su , C. Chen , J. Electroanal. Chem. 2025, 984, 119060.

[58] G. Zhao , T. Xu , Y. Zhao , Z. Yi , L. Xie , F. Su , Z. Yao , X. Zhao , J. Zhang , W. Xie , X. Li , L. Dong , C.‐M. Chen , Energy Storage Mater. 2024, 67, 103282.

[59] D. Sun , L. Zhao , P. Sun , K. Zhao , Y. Sun , Q. Zhang , Z. Li , Z. Ma , F. Zheng , Y. Yang , C. Lu , C. Peng , C. Xu , Z. Xiao , X. Ma , Adv. Funct. Mater. 2024, 34, 2403642.

[60] R. Xu , N. Sun , H. Zhou , X. Chang , R. A. Soomro , B. Xu , Battery Energy 2023, 2, 20220054.

[61] H. Chen , N. Sun , Q. Zhu , R. A. Soomro , B. Xu , Adv. Sci. 2022, 9, 2200023. 10.1002/advs.202200023 PMC9284145 35508900

[62] D. Chen , K. Luo , Z. Yang , Y. Zhong , Z. Wu , Y. Song , G. Chen , G. Wang , B. Zhong , X. Guo , Carbon 2021, 173, 253.

[63] L. Ji , Y. Zhao , L. Cao , Y. Li , C. Ma , X. Qi , Z. Shao , J. Mater. Chem. A 2023, 11, 26727.

[64] L. Kitsu Iglesias , S. D. Marks , N. Rampal , E. N. Antonio , R. de Ferreira de Menezes , L. Zhang , D. Olds , S. E. Weitzner , K. G. Sprenger , L. F. Wan , M. F. Toney , Small 2025, 21, 2505561. 10.1002/smll.202505561 40442960

[65] J. Zhao , X.‐X. He , W.‐H. Lai , Z. Yang , X.‐H. Liu , L. Li , Y. Qiao , Y. Xiao , L. Li , X. Wu , S.‐L. Chou , Adv. Energy Mater. 2023, 13, 2300444.

