# Supplementary material for: Carboxyl‐Guided Ultrafine Zinc‐Acetate Templating Enables Closed‐Pore Engineering in Coal‐Derived Hard Carbon Anodes for High‐Performance Sodium‐Ion Batteries
Source: Adv Sci (Weinh). 2025 Oct 13;13(1):e12483. doi: 10.1002/advs.202512483 (PMC12767055; doi:10.1002/advs.202512483)
Supplement: Supplementary file 1 — Supporting Information [file ADVS-13-e12483-s001.docx]

Supporting Information

**Carboxyl-induced Ultrafine Zinc Acetate Templating Strategy Enabling Closed-pore Engineering for Coal-Based Hard Carbon Anode of High-Performance Sodium Ion Batteries**

*Jialiang Yuan, Guokan Liu, Chi Wang , Fang Wan , Lang Qiu , Bo Yuan, Tingru Chen*, Zhenguo Wu**

J. Yuan, G. Liu, C. Wang, Prof. F. Wan, Dr. L. Qiu, Dr. T Chen, Prof. Z. Wu.

School of Chemical Engineering, Sichuan University, Chengdu 610065, P. R. China.

E-mail: chentingru@scu.edu.cn, zhenguowu@scu.edu.cn

B. Yuan

Ordos Carbon Neutral Research and Application Co., Ltd., Ordos City 017010, P.R. China

**Experiments**

*Material Synthesis*：Bituminous coal, originating from Erdos, contains a significant amount of impurities. After being crushed and sieved through a 100-mesh screen, it undergoes an impurity removal treatment using 36% hydrochloric acid and 40 wt% hydrofluoric acid. Subsequently, BC is mixed with sodium hydroxide in a 1:2 weight ratio in a container, and water is added to dissolve the NaOH (water: NaOH = 7:3 wt). The mixture is stirred at 60 °C for 12 hours, centrifuged, washed with water, and then dried to obtain ABC. A certain mass of ZA is weighed, dissolved in water, and evenly mixed with ABC. The mixture is then dried at 60 °C for 2 hours to obtain ABC loaded with ZA. A certain mass of PR is weighed, dissolved in ethanol, and used to soak the ABC loaded with ZA. The soaked ABC is then dried at 60 °C for 12 hours. The sample is subsequently placed in a tubular furnace and pre-oxidized at 150 °C in an air atmosphere for 10 hours to obtain the precursor material. The precursor is pre-calcined at 700 °C in an argon atmosphere at a heating rate of 4 °C min^-1^ for 2 hours. After being removed, it is thoroughly washed with 36% hydrochloric acid to remove zinc oxide, washed with water until neutrality, and dried. Finally, the precursor is placed in a tubular furnace and calcined at 1400 °C in an argon atmosphere at a heating rate of 4 °C min^-1^ for 2 hours to obtain the final HC material.

*Material Characterization:* The morphology and microstructure of the prepared samples were characterized by scanning electron microscopy (SEM, JSM 7610F) and high-resolution transmission electron microscopy (HRTEM, JEM-F200, Japan). The surface elements of the material were analyzed by Thermo Scientific K-Alpha (USA) using X-ray photoelectron spectroscopy (XPS). The gas production of the precursor during calcination was characterized by TG-IR (STA449F5-INVENIOR). The disorder and crystalline properties of the materials were characterized using a 532 nm Raman spectrometer (Renishaw inVia-Reflex, UK) and X-ray diffraction (XRD, X'Pert Pro, PANalytical B.V.) with 40 kV and 40 mA Cu Kα radiation. The pore structure of HC was characterized at 77 K using N_2_ adsorption/desorption analysis (N_2_ adsorption/desorption analysis) from Micromeritics (ASAP 2460), USA. The closed pores were studied using a small-angle X-ray scattering device (French Xenocs Xeuss 3.0). Fourier transform infrared spectroscopy (FTIR) (Nicolet iS 10) was used to study the chemical composition of the material.

*Electrochemical Performance*: All electrochemical tests were performed in a CR2032 button battery assembled in a glove box filled with argon gas (H_2_O< 0.2 ppm, O_2_< 0.2 ppm). The working electrode is composed of active material, conductive carbon black, and sodium alginate with a mass ratio of 9.5:0.25:0.25. The payload mass of each pole plate is 1.5 mg cm^-2^. 1.0 M NaPF_6_ was dissolved in ethylene glycol dimethyl ether (DME) as an electrolyte. Whatman GF/D glass fiber as a diaphragm. The electrochemical performance was tested by the NEWARE CT-3008W battery tester. The voltage range is 0‒3 V, and the charge and discharge test is carried out at 30 ℃. The constant current intermittent titration technique (GITT) is performed at a current of 20 mA g^-1^, and the relaxation time is set to 1h. Scanning cycle voltammetry (CV) tests were performed on the CHI730E electrochemical workstation at various scanning speeds and voltages ranging from 0 to 3 V. The full battery assembly uses the HC derived from ABC-PR-ZAX(20%) as the anode, Na_3_V_2_(PO_4_)_3_ as the cathode, and the N/P ratio is 1.15:1. The electrolyte was dissolved with 1 M NaClO_4_ in a 1:1(volume ratio) mixture of vinyl carbonate (EC) and diethyl carbonate (DEC) and added with 5.0 vol % vinyl fluorocarbonate (FEC). The full battery charge and discharge voltage range is 1.5- 3.8V.


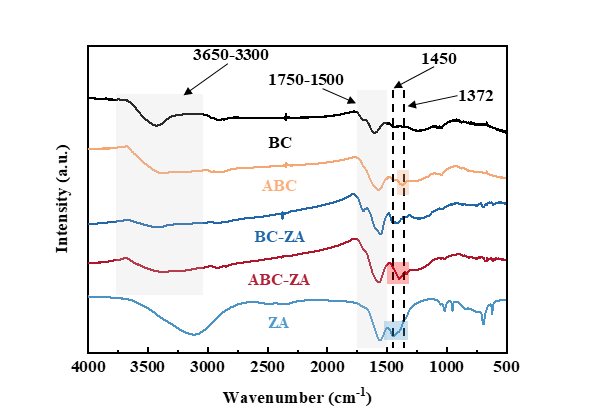


**Figure S1.** The FTIR spectra of BC, ABC, BC-ZA, ABC-ZA and ZA.

**
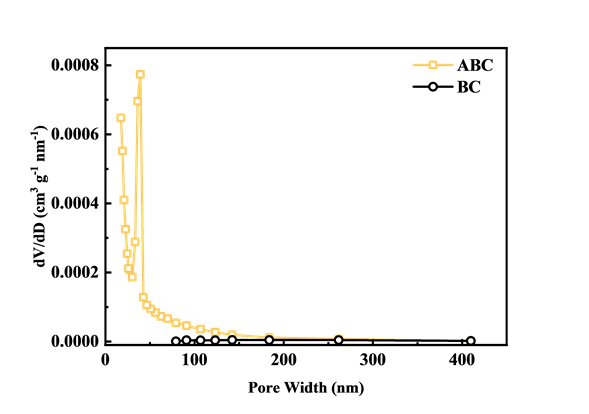
**

**Figure S2.** The pore size distribution diagrams of BC and ABC.


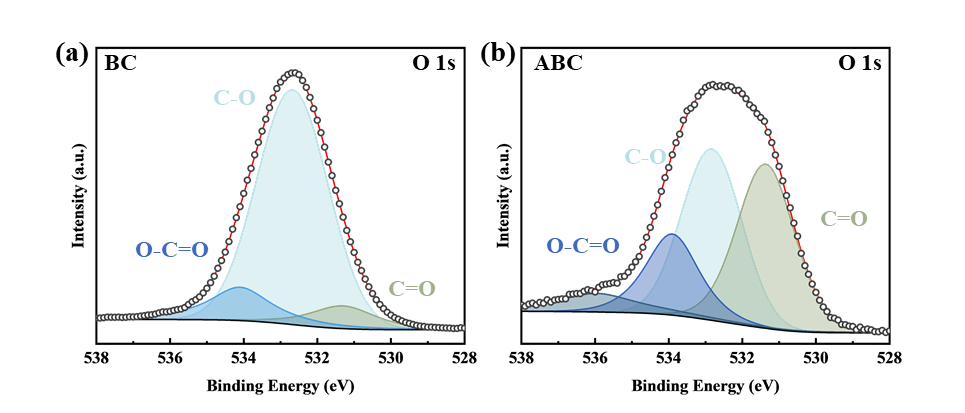


**Figure S3.** (a, b) Peak fitting of XPS O 1s for BC and ABC.

**
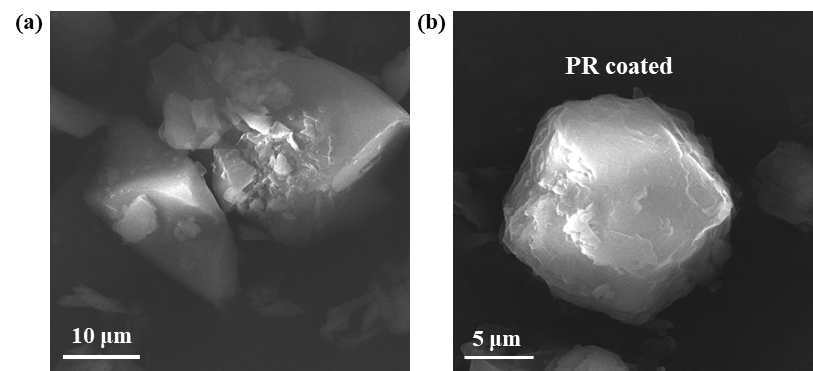
**

**Figure S4.** (a) SEM image of phenolic resin. (b) SEM image of the precursor after encapsulation of phenolic resin.

**
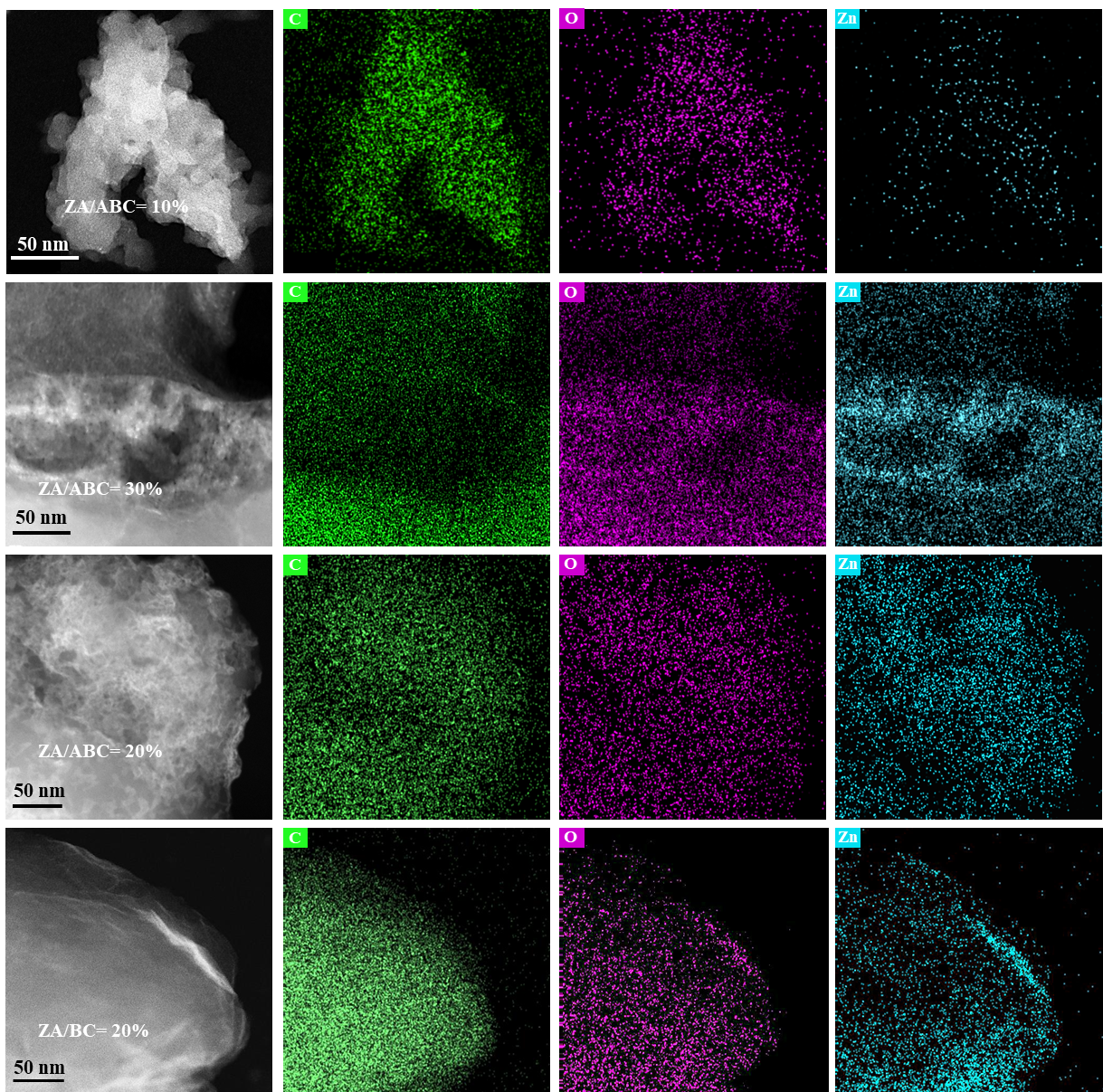
**

**Figure S5.** The changes in the number of sp²-hybridized carbon atoms and the transformation from quadrilateral to octagonal polygons during the carbonization process were studied through molecular dynamics simulation time.


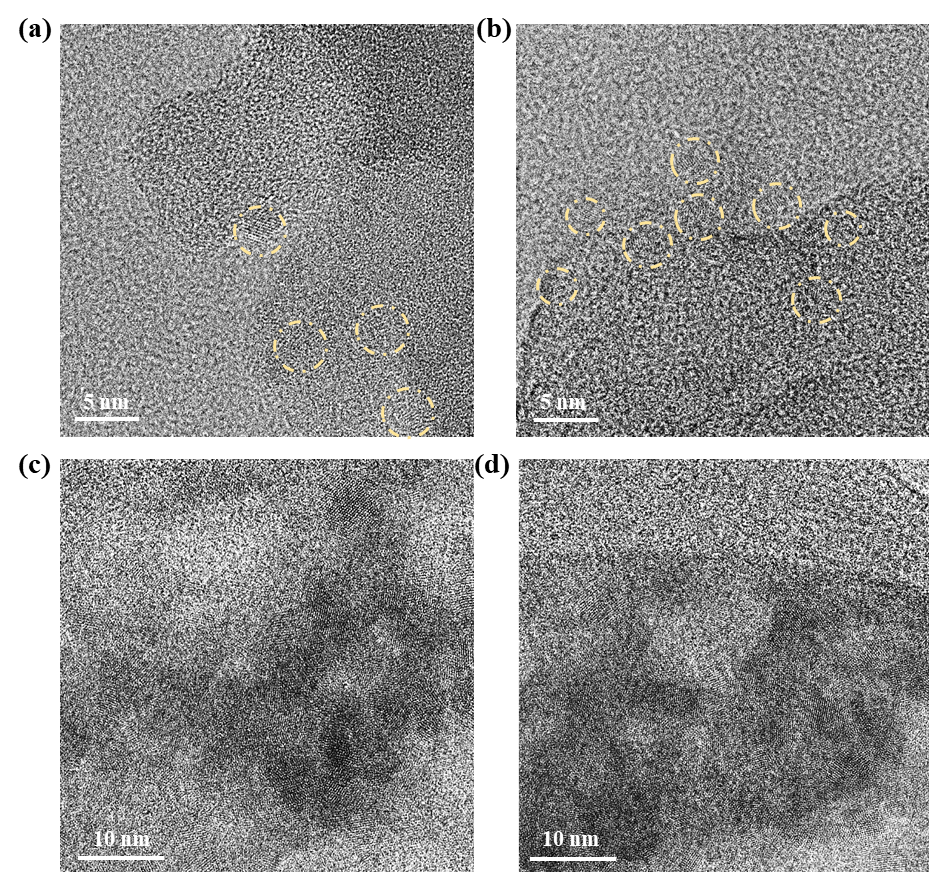


**Figure S6.** (a, b) HRTEM images of 10% ZA particles loaded on ABC. (c, d) HRTEM images of 30% ZA particles loaded on ABC.


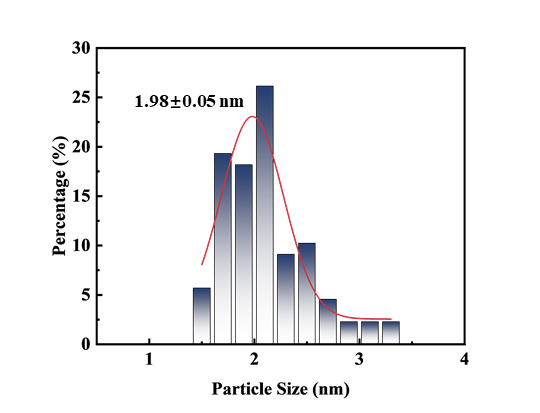


**Figure S7.** The particle size of the ZA component loaded at 20% on ABC.


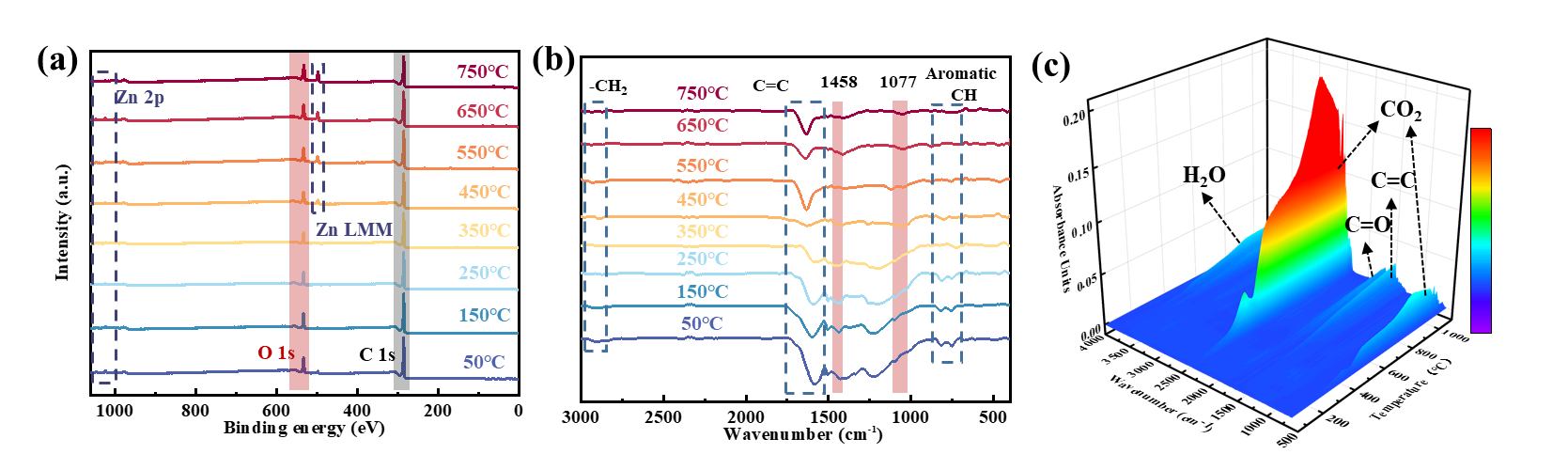


**Figure S8.** (a) XPS spectra of ABC-PR-ZAX(20%) at different pyrolysis temperatures. (b) FTIR curves of ABC-PR-ZAX(20%) at different pyrolysis temperatures. (c) Thermogravimetric infrared (TG-IR) image of ABC-PR-ZAX(20%).

In Figures S8a and b, the curve at 50°C corresponds to the unoxidized precursor (ABC-PR-ZA), while the curve at 150°C represents the precursor after air oxidation (ABC-PR-ZAX), followed by pyrolysis under inert atmosphere. In the sample at 450°C, a distinct spectral peak appears at approximately 500 eV, which is attributed to the zinc Auger peak in ZnO. During the pyrolysis process, the more obvious absorption peaks of coal and phenolic resin include the asymmetric stretching vibration absorption peak of aliphatic CH_2_ at 2920 cm^-1^, the stretching vibration absorption peak of aromatic C=C at 1590 cm^-1^, and the absorption peak in the range of 1510 to 754 cm^-1^. These peaks gradually weaken with the increase of pyrolysis temperature, reflecting the loss of volatile components in coal and phenolic resin. Among them, at higher temperatures (550 to 750°C), a large number of methylene groups in phenolic resin break and release phenol and various derivatives. Meanwhile, the unstable components in coal also undergo significant pyrolysis, which corresponds to the significant increase in the intensity of various gas component absorption peaks after 500°C in Figure S8c.


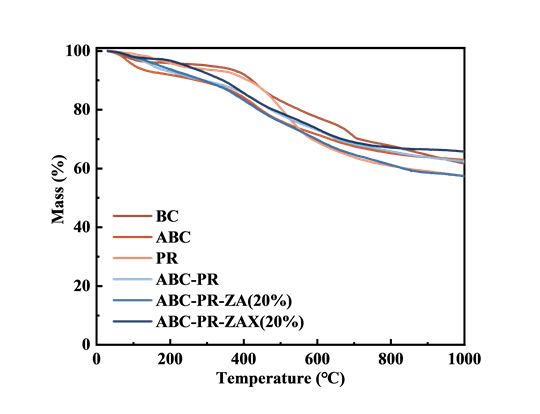


**Figure S9.** The TG curves of BC, ABC, PR, ABC-PR, ABC-PR-ZA (20%), and ABC-PR-ZAX (20%).

**
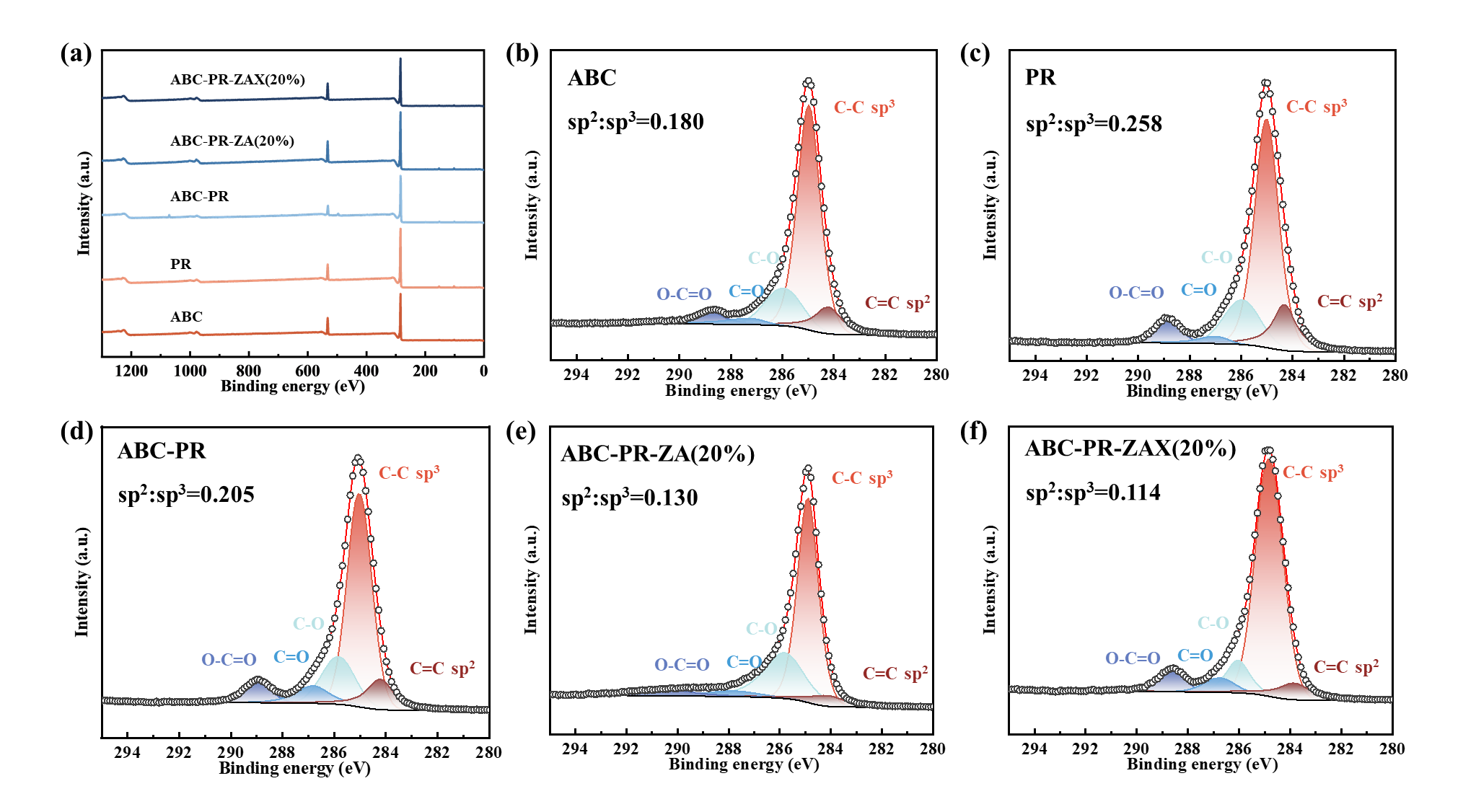
**

**Figure S10.** (a) The full XPS spectrum of HC derived from ABC, PR, ABC-PR, ABC-PR-ZA(20%), ABC-PR-ZAX(20%). (b-f) Peak fitting of XPS C 1s of HCs.

**
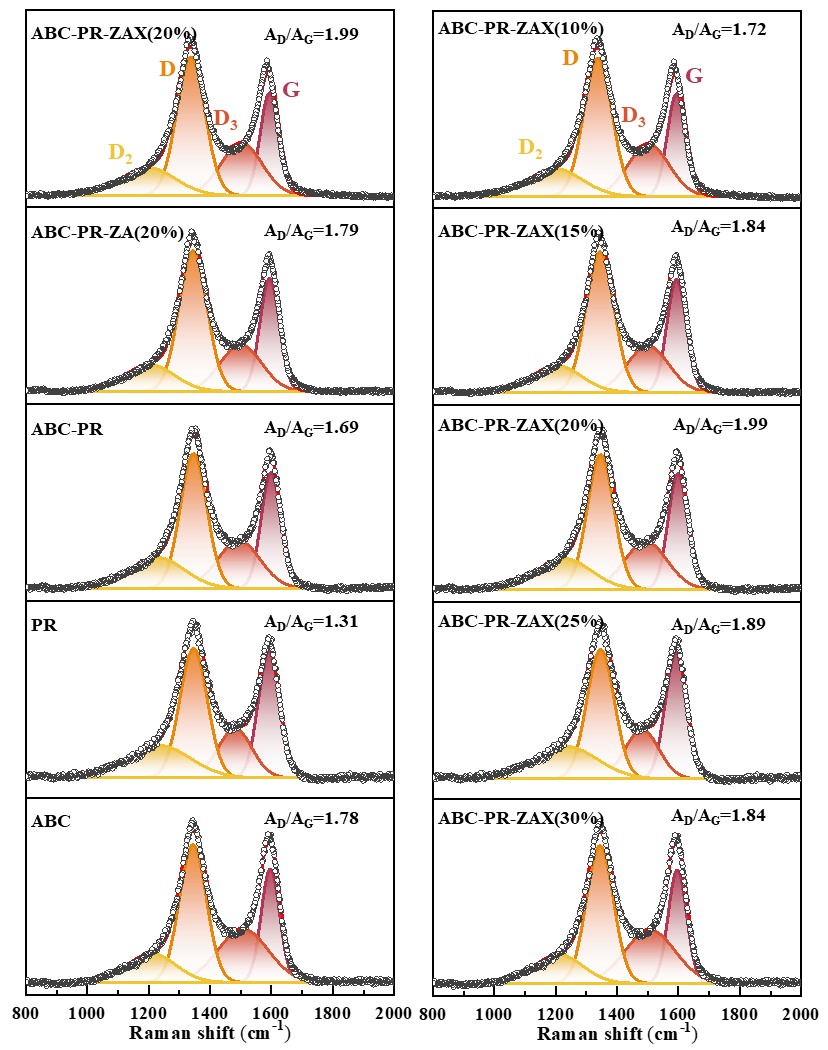
**

**Figure S11.** Raman spectra fitting diagrams of HC derived from different precursors.

**
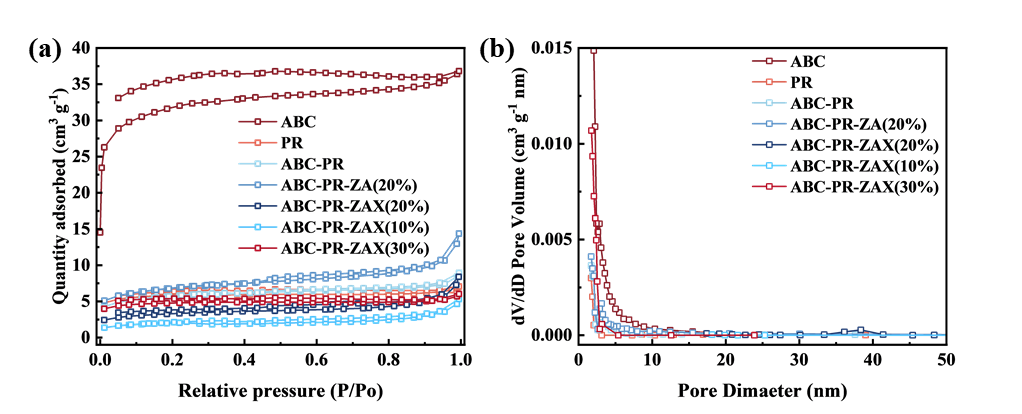
**

**Figure S12.** (a, b) Nitrogen isothermal adsorption-desorption isotherms and pore size distribution diagrams of HCs derived from different precursors.

**
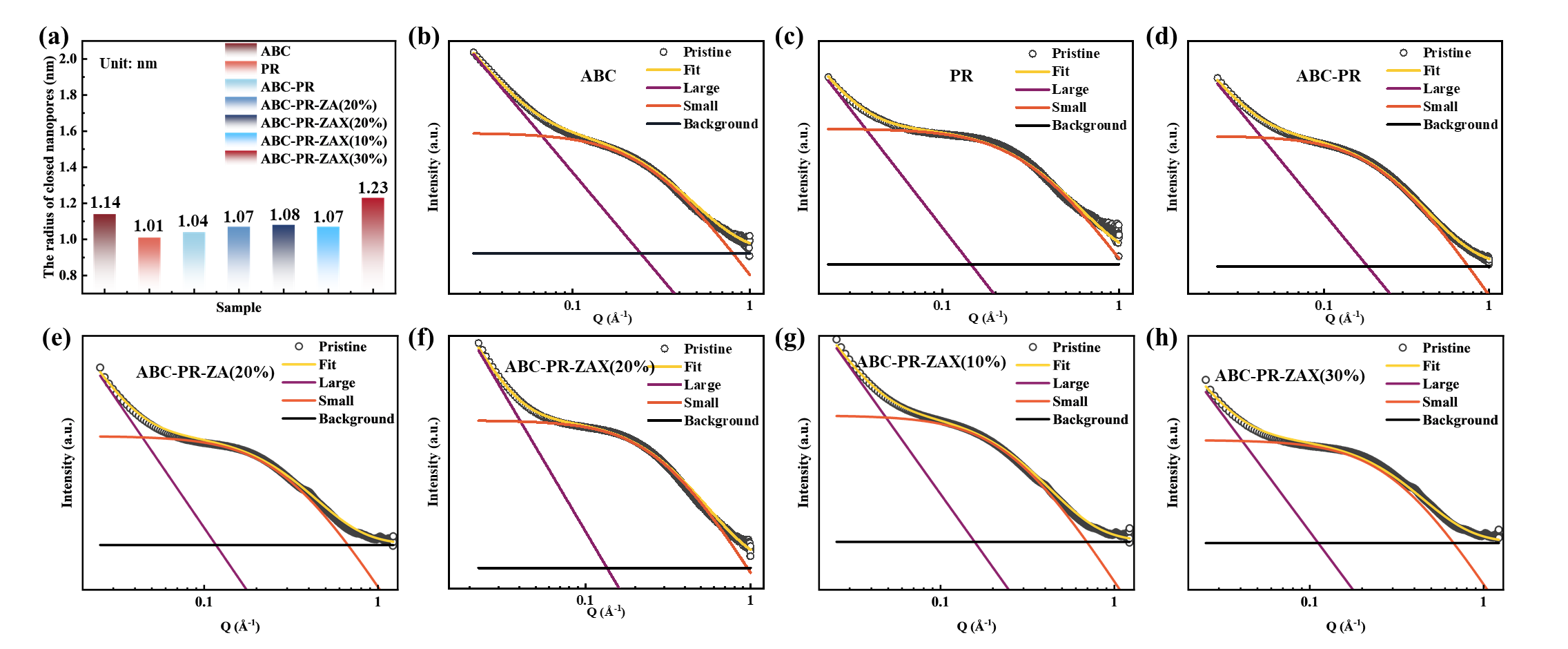
**

**Figure S13.** The closed pore diameters of HCs derived from different precursors and the SAXS fitting curves.

**
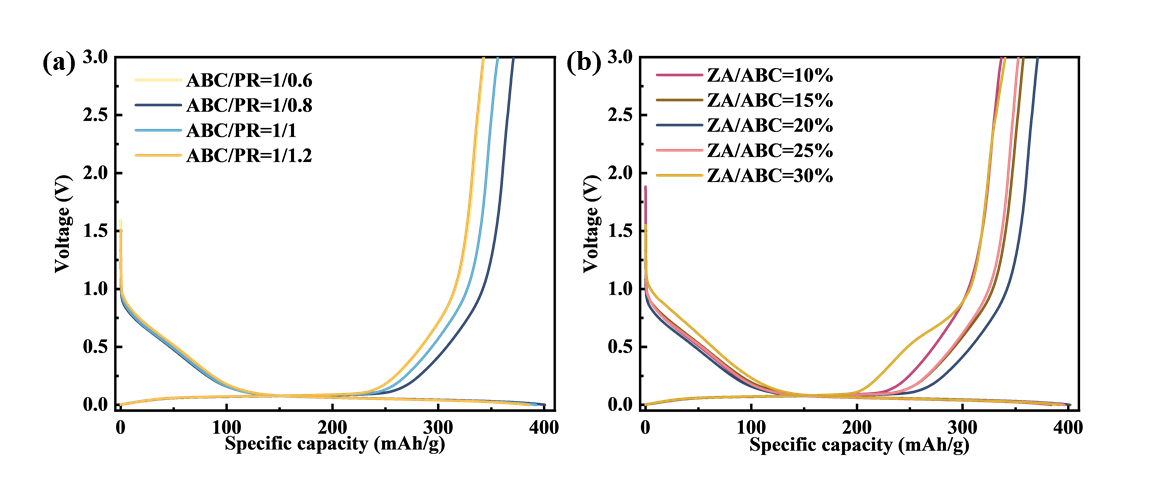
**

**Figure S14.** (a) The first charge-discharge curves of HCs prepared under different ABC and PR ratios with a fixed ZA load of 20%. (b) Fixed ABC: PR = 1:0.8 (wt), the first charge-discharge curves of HCs prepared under different ZA loading conditions.

**
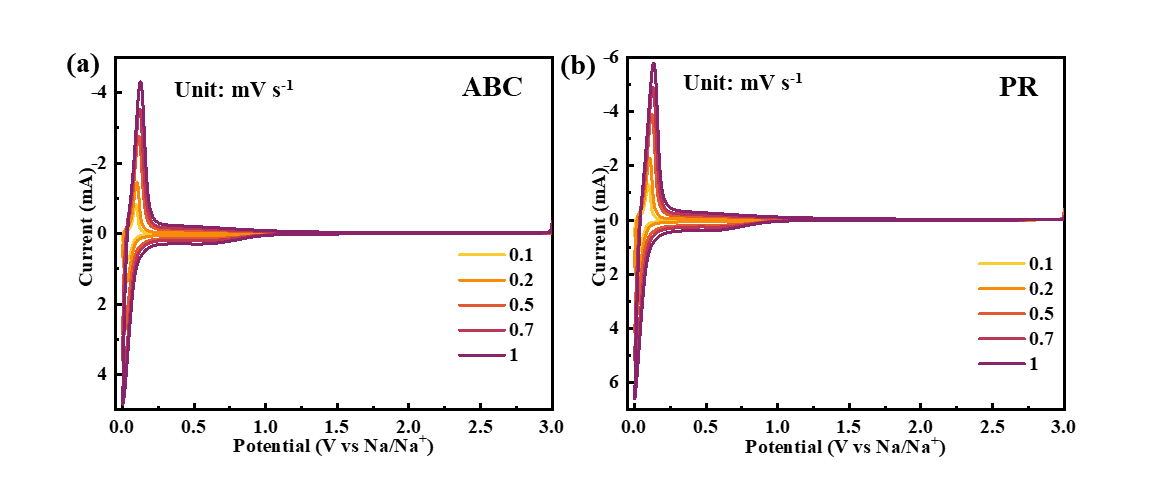
**

**Figure S15.** (a, b) Cyclic voltammogram of ABC and PR derived HCs at different scan rates.

**
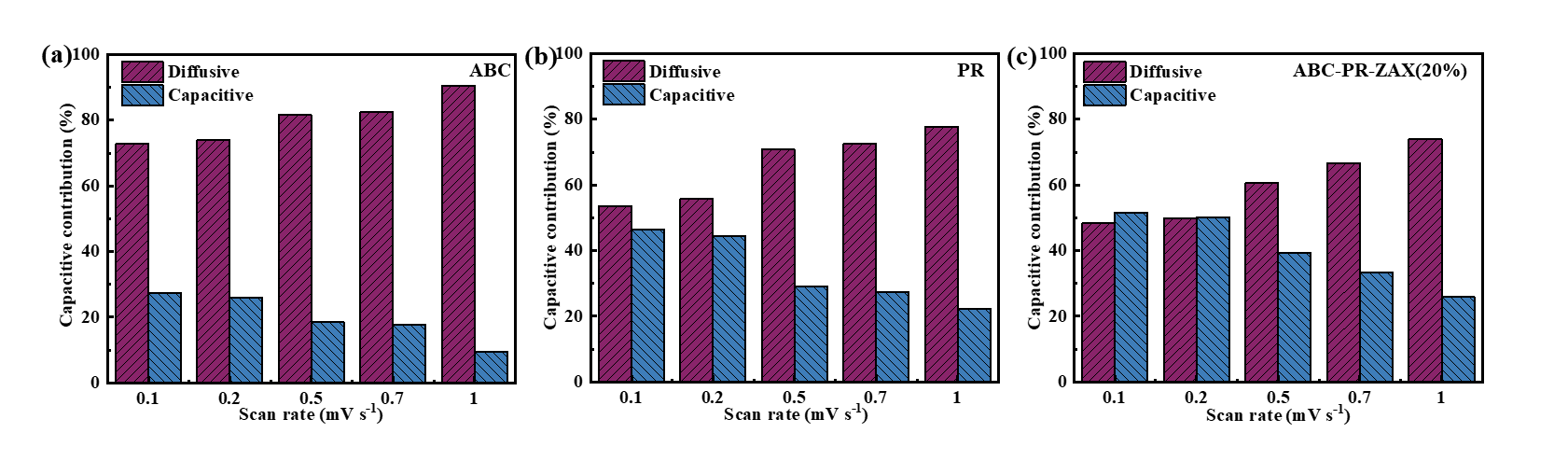
**

**Figure S16.** (a-c) Capacitance contribution of ABC, PR, ABC-PR-ZAX(20%) derived HCs at different voltage sweep rates.

**
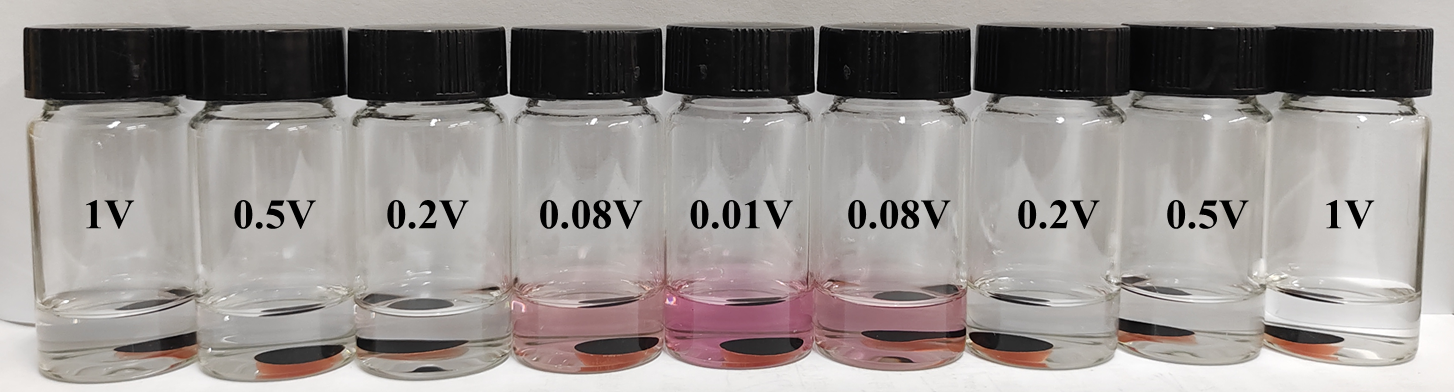
**

**Figure S17.** The color change of phenolphthalein ethanol solution after reacting with electrodes of different potentials.

**Table S1.** Component analysis and elemental analysis of bituminous coal.

| Moisture  (wt%) | Ash  (wt%) | Volatile  component(wt%) | Fixed  carbon(wt%) | C  (wt%) | H  (wt%) | O  (wt%) | N  (wt%) | S  (wt%) |
| --- | --- | --- | --- | --- | --- | --- | --- | --- |
| 6.79 | 6.21 | 36.06 | 55.94 | 76.638 | 5.372 | 17.243 | 0.677 | 0.070 |

**Table S2.** Pore structure parameters of HCs.

| Samples | True density  g cm^-3^ | S_SAXS_  cm^2^ g^-1^ |
| --- | --- | --- |
| ABC | 1.97 | 171.41 |
| PR | 1.9 | 249.02 |
| ABC-PR | 1.83 | 299.91 |
| ABC-PR-ZA(20%) | 1.58 | 533.92 |
| ABC-PR-ZAX(20%) | 1.56 | 551.52 |
| ABC-PR-ZAX(10%) | 1.72 | 389.48 |
| ABC-PR-ZAX(30%) | 1.69 | 363.99 |

**Table S3.** Various data of HC derived from ABC-PR-ZAX(20%).

| Sample | ABC-PR-ZAX(20%) |
| --- | --- |
| Specific capacity (mAh g^-1^) | 370.96 |
| ICE (%) | 92.5 |
| Specific surface area (m^2^ g^-1^) | 24.76 |
| Ash content (%) | 0.25 |
| Hydration (%) | 0.21 |
| Compaction density (g cm^-3^) | 1.04 |

**Table S4.** Carbonization yield of different materials.

| Sample | Carbonization yield (%) |
| --- | --- |
| BC | 52.3 |
| ABC | 59.1 |
| PR | 48.0 |
| ABC-PR | 58.4 |
| ABC-PR-ZA  ABC-PR-ZAX | 50.6  60.3 |
